# Structure of DNA-CMG-Pol epsilon elucidates the roles of the non-catalytic polymerase modules in the eukaryotic replisome

**DOI:** 10.1038/s41467-018-07417-1

**Published:** 2018-11-29

**Authors:** Panchali Goswami, Ferdos Abid Ali, Max E. Douglas, Julia Locke, Andrew Purkiss, Agnieszka Janska, Patrik Eickhoff, Anne Early, Andrea Nans, Alan M. C. Cheung, John F. X. Diffley, Alessandro Costa

**Affiliations:** 1 Macromolecular Machines Laboratory, The Francis Crick Institute, 1 Midland Road, London, NW1 1AT UK; 2 Chromosome Replication Laboratory, The Francis Crick Institute, 1 Midland Road, London, NW1 1AT UK; 3 Structural Biology Science Technology Platform, The Francis Crick Institute, 1 Midland Road, London, NW1 1AT UK; 4 Department of Structural and Molecular Biology, Institute of Structural and Molecular Biology, University College London, London, UK; 5 Institute of Structural and Molecular Biology, Biological Sciences, Birkbeck College, London, WC1E 7HX UK

## Abstract

Eukaryotic origin firing depends on assembly of the Cdc45-MCM-GINS (CMG) helicase. A key step is the recruitment of GINS that requires the leading-strand polymerase Pol epsilon, composed of Pol2, Dpb2, Dpb3, Dpb4. While a truncation of the catalytic N-terminal Pol2 supports cell division, Dpb2 and C-terminal Pol2 (C-Pol2) are essential for viability. Dpb2 and C-Pol2 are non-catalytic modules, shown or predicted to be related to an exonuclease and DNA polymerase, respectively. Here, we present the cryo-EM structure of the isolated C-Pol2/Dpb2 heterodimer, revealing that C-Pol2 contains a DNA polymerase fold. We also present the structure of CMG/C-Pol2/Dpb2 on a DNA fork, and find that polymerase binding changes both the helicase structure and fork-junction engagement. Inter-subunit contacts that keep the helicase-polymerase complex together explain several cellular phenotypes. At least some of these contacts are preserved during Pol epsilon-dependent CMG assembly on path to origin firing, as observed with DNA replication reconstituted in vitro.

## Abstract (web summary)

Eukaryotic origin firing depends on assembly of the Cdc45-MCM-GINS (CMG) helicase, which requires the leading-strand polymerase Pol ɛ. Here the authors present a structural analysis of a CMG Pol ɛ on a DNA fork, providing insight on the steps leading productive helicase engagement to the DNA junction.

## Introduction

DNA replication requires tight coordination between DNA unwinding and synthesis within the replisome^1^. In eukaryotic cells, the replisome is assembled in three distinct steps leading to origin licensing, DNA untwisting, and replication fork establishment^2–5^. First, the minichromosome maintenance protein complex (MCM) helicase, a ring-shaped ATPase, is loaded onto origins of replication as an inactive double hexamer that encircles duplex DNA^6–9^, in a process that involves ATP hydrolysis by MCM^10,11^ and requires loading factors ORC, Cdc6, and Cdt1^12^. Second, helicase activators Cdc45 and GINS are recruited in a regulated manner, mediated by targets of cyclin-dependent kinase (CDK) phosphorylation, Sld2 and Sld3, and by the replisome-maturation scaffolds, Dpb11 and Sld7. MCM phosphorylation by DDK allows Cdc45-Sld3-Sld7 binding to the double hexamer, dependent on phospho-MCM recognition by Sld3^13–18^. GINS is recruited onto the MCM together with the leading-strand polymerase Pol epsilon, phospho-Sld2 and Dpb11, together forming the pre-loading complex^19^. Assembly of a stable Cdc45-MCM-GINS (CMG) holo-helicase requires a change in the MCM ATPase state, with release of ADP and binding of ATP, concomitantly promoting separation of the double hexamer into single hexamers and untwisting of duplex DNA^2^. The third step in origin activation is replication fork establishment, which depends on the recruitment of additional firing factors Mcm10, RPA, and Pol alpha^13,20^.

The organizing center of the replisome is the MCM^21^, made of six homologous polypeptides that all share the same domain organization. MCM proteins form an N-terminal duplex DNA-interacting collar and a AAA+ (ATPase associated with various cellular activities) tier, featuring bipartite active sites with catalytic residues contributed by neighboring subunits^22^. Work on *Drosophila* CMG revealed that the helicase motor is functionally asymmetric, as certain ATPase centers (Mcm2-5 and Mcm5-3) are strictly required for DNA unwinding, while other sites (Mcm6-4 and Mcm4-7) can be inactivated with minimal effect on helicase activity^23^. Electron microscopy (EM) studies of both *Drosophila* and yeast CMG have revealed that Cdc45 and GINS (Sld5, Psf1, Psf2, Psf3) bind to the side of the MCM ring by engaging the N-terminal tier of MCM and stabilizing the Mcm2-5 and Mcm5-3 interfaces, respectively^24–26^.

Reconstitution studies showed that the leading-strand polymerase Pol epsilon forms a stable complex with the CMG^21,27^, by binding to the ATPase tier of MCM via a non-catalytic domain^28^. Hetero-tetrameric Pol epsilon plays a key role in replisome maturation and origin activation^19,29,30^. In this protein assembly, Dpb3 and Dpb4 are ancillary, DNA-binding subunits containing a histone fold^31^. Pol2 is the catalytic subunit, with the N-terminal half containing DNA synthesis/exonuclease functions^32^. The C-terminal half of Pol2 (C-Pol2) has been predicted to contain a second polymerase fold, which has become inactivated during evolution^33^, and is followed by a zinc-finger appendix^34^. Notably, the catalytic domain of Pol epsilon is dispensable for viability (though cells are sick), while the non-catalytic C-Pol2 is essential^35,36^. Dpb2, the second largest subunit of Pol epsilon, is also essential for viability and contains an inactivated calcineurin-like exonuclease fold^34^ decorated by an N-terminal appendix structurally related to the AAA+ ATPase lid domain^37^. It is clear that non-catalytic modules in Pol epsilon are required for helicase activation and origin firing^13,35,38^, although the molecular basis is poorly understood. Furthermore, what role these inactivated domains play during fork progression is unclear^1^. To explain the functions of the leading-strand polymerase during DNA replication, we determined the structure of the non-catalytic modules of Pol epsilon by cryo-EM and biochemically assayed their DNA-binding properties. We have also determined the structure of Pol epsilon bound to a DNA-fork-engaged CMG complex to gain insights into the architecture of the replisome during fork progression. We found that non-catalytic Pol epsilon causes a conformational change within the MCM ATPase, affecting the CMG interaction with the replication fork nexus. Based on predictions from the DNA-CMG-Pol epsilon structure, we identified the minimal complement of Pol epsilon modules required for assembling the CMG, by using the in vitro reconstituted DNA replication system. Therefore, certain protein–protein interactions important for CMG assembly during origin activation appear to be preserved in the CMG-Pol epsilon complex reconstituted on the replication fork. Together, our data provide important insights into the distinct roles of Pol epsilon during DNA replication.

## Results

### Structure of the Pol epsilon modules essential for viability

**Fig. 1:**
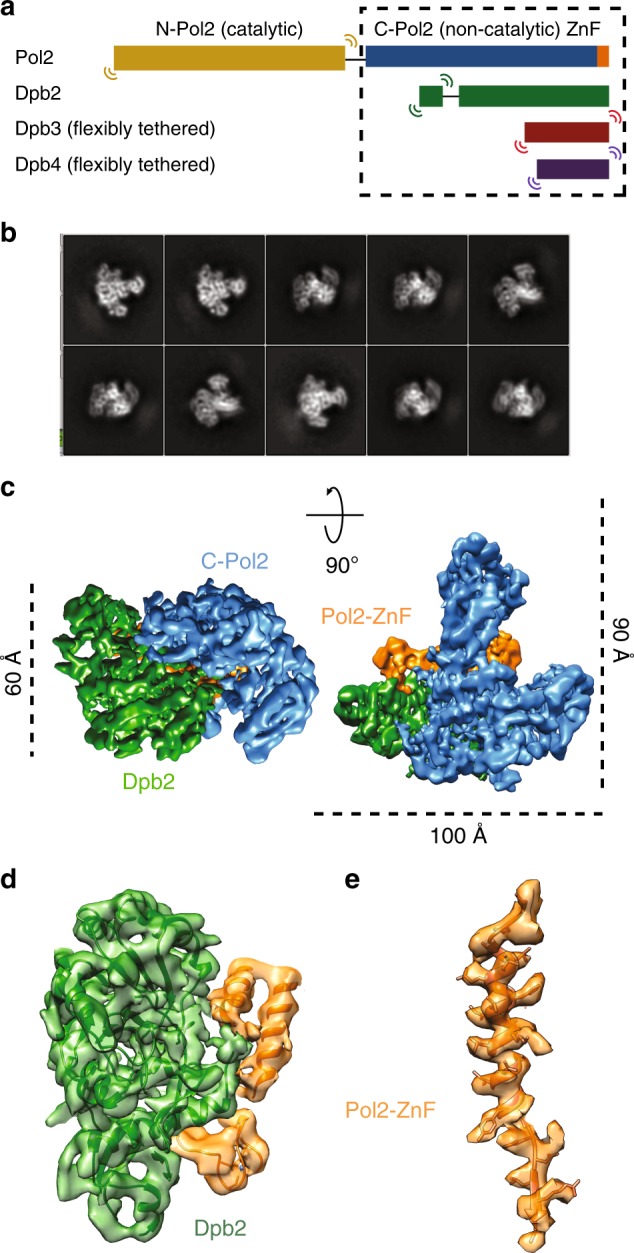
Cryo-EM structure of the tetrameric Pol epsilon complex, lacking the catalytic domain (deltacat). **a** Subunit composition and domain organization of yeast Pol epsilon. N-Pol2 stands for N-terminal Pol2. C-Pol2 stands for C-terminal Pol2. ZnF stands for zinc-finger appendix. **b** 2D class averages of deltacat. **c** Surface rendering of the deltacat structure solved to 4.45 Å resolution. Dpb2 is green, Pol2 catalytically dead polymerase fold is blue, and Pol2 zinc-finger appendix is orange. **d** Atomic model for the yeast Dpb2 (green) bound to the Pol2 C-terminal zinc-finger appendix (orange), built into the cryo-EM map. **e** Detail of the Pol2 zinc-finger appendix (further map sharpening with phenix.auto_sharpen)

**Fig. 2:**
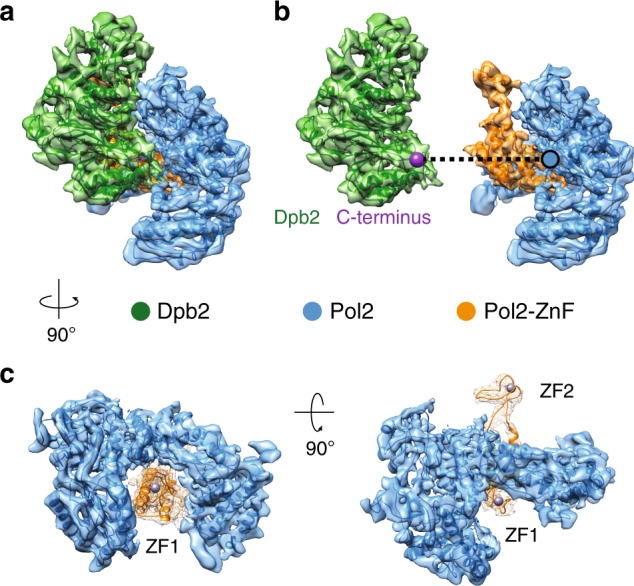
Interaction between C-Pol2 and Dpb2. **a** Atomic model of C-Pol2 and Dpb2 built into the cryo-EM map. **b** Dpb2 C-terminus is poised towards the C-Pol2 interface, explaining why a truncation of the Dpb2 C-terminal region results in a lethal phenotype. **c** Pol2 zinc-finger appendix resides in the core of the Pol2 polymerase fold. This explains why a point mutation disrupting ZF1 is not compatible with viability. A second zinc-finger motif (ZF2) projects from the core of the complex

To understand the role of the essential Pol epsilon modules during DNA replication, we studied the structure of a truncation mutant of the yeast tetrameric complex, lacking the dispensable, flexibly tethered N-terminal catalytic domain of Pol2^28^ (hereafter, deltacat, Fig. 1a). Our first attempts to trap particles in vitreous ice resulted in severe aggregation, probably as a consequence of particle exposure to the air–water interface. To mitigate this problem, we incubated our preparation with 0.01% of crosslinking agent glutaraldehyde for one minute on ice, before cryo-grid making (Supplementary Fig. 1). The resulting particles appeared homogeneous and monodisperse. We deemed this preparation suitable for image acquisition on a Titan Krios EM equipped with a Falcon III direct electron detector operated in counting mode. Given the relatively small size of our protein target and inherent flexibility detected in preliminary cryo-EM characterization^28^, we acquired a dataset at low defocus and using the Volta phase plate (VPP). As image contrast in these conditions is dramatically improved, high-resolution structures can be obtained using significantly fewer particles compared to defocus-based phase-shift imposition. This strategy provides an important advantage when dealing with inherently flexible particles^39^. Two-dimensional averages showed high-resolution features for several particle orientations, with one prominent view revealing an anchor-shaped structure (Fig. 1b and Supplementary Fig. 1). A poorly resolved feature could be detected and deeper 2D classification efforts revealed a structured domain flexibly tethered to the particle core (Supplementary Movie 1). We determined a 3D structure of the deltacat core based on 161,372 particles, to an average resolution of 4.45 Å (4.3 Å in the core and 5 Å in the periphery), revealing a compact assembly of dimensions 100 × 90 × 60 Å (Fig. 1c and Supplementary Fig. 1-2). To interpret the map, we have docked a recently published structure of human C-terminal Dpb2 bound to the Pol2 C-terminal zinc-finger appendix^34^, providing an unambiguous fit. A yeast homology model based on the human structure was subsequently used as a template for real-space refinement (Fig. 1d–e). The residual density displayed an obvious resemblance to a DNA polymerase fold and homology searches performed with HHpred^40 ^indicated that the DNA polymerase domain of Pol1, from the tetrameric Pol alpha/primase complex^41^, is a suitable model for C-Pol2 (Fig. 2a). Docking of a Pol1-based homology model of C-Pol2 into the cryo-EM density required splitting the polymerase into three domains, which were used in independent rigid-body fitting and subsequent real-space refinement. The resulting structure shows a polymerase fold with jaws spread wide open. These data provide the first structural evidence that C-Pol2 contains a DNA polymerase fold^33^ (Supplementary Movie 2). In agreement with the notion that certain elements are only flexibly tethered to the deltacat core, no density was retrieved for the N-terminal domain of Dpb2, nor for the histone-like subunits Dpb3-Dpb4. We speculated at this stage that at least one of these elements might become structured once Pol epsilon engages other replisome partner proteins (further discussed below). Our structure of the Dpb2/C-Pol2 interaction core explains key Pol epsilon phenotypes. For example, the C-terminus of Dpb2 intimately contacts C-Pol2, explaining why a truncation of the last six amino acids in Dpb2 prevents Dpb2 binding to Pol2-Dpb3-Dpb4 and results in a lethal phenotype^42^ (Fig. 2b). Likewise, a so-called ZnF1 element in the Pol2 zinc-finger appendix emerges as the organizing center of the deltacat core, explaining why cysteine-to-alanine single amino acid changes in this region can alter the essential non-catalytic Pol epsilon core, hence abolishing cellular growth (Fig. 2c)^35^.

### DNA binding by Pol epsilon

**Fig. 3:**
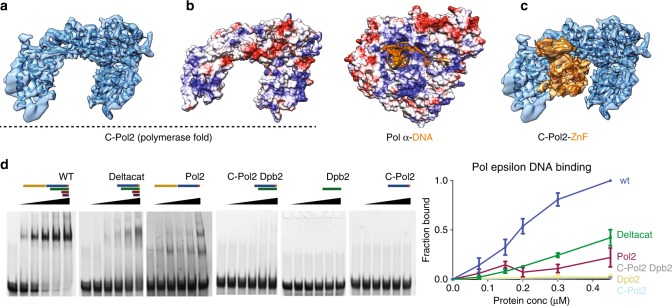
The C-terminal half of Pol2 contains a catalytically dead polymerase fold that has lost its DNA-binding function. **a** C-Pol2 contains an inactive polymerase fold with jaws wide open. **b** Coulombic surface coloring of the C-Pol2 catalytically dead polymerase reveals a lack of positive charges in the vestiges of the DNA-binding groove, while the DNA-binding site in the related Pol alpha catalytic domain is positively charged. **c** The DNA-binding groove in C-Pol2 is occupied by the Pol2 zinc-finger appendix. **d** DNA-binding assay with various Pol epsilon variants. Only constructs containing the N-Pol2 catalytic domain or the histone-related proteins Dpb3-Dpb4 bind to DNA. We were unable to detect any DNA binding for C-Pol2 and Dpb2. On the right, quantification of the DNA-binding assays. Each experiment was repeated three times and error bars indicates standard deviation. Also refer to Supplementary Fig. 3

If C-Pol2 and Dpb2 are non-catalytic modules that evolved from DNA-processing enzymes^43^, we reasoned that these domains might have retained their DNA-binding function. The DNA-binding domain of Pol alpha subunit Pol1 has been co-crystallized with DNA^41^. We therefore asked whether the open polymerase configuration found in C-Pol2 could still retain DNA-binding capabilities. Structure superposition revealed no obvious steric clashes between the open C-Pol2 polymerase fold and a primer–template junction. However, coulombic surface coloring indicates that the C-Pol2 polymerase domain lacks the positively charged region seen in DNA-binding grooves of other DNA polymerases. Furthermore, inspecting a more complete Pol2 structure that extends beyond the conserved polymerase module revealed that the C-terminal zinc-finger appendix occludes the DNA-binding groove in the dead polymerase fold (Fig. 3a–c). Admittedly, such tight polymerase–zinc-finger interaction might have been stabilized by the crosslinking agent used in our preparation. Likewise, this configuration might change in larger complexes such as full-length Pol epsilon or in a helicase–polymerase assembly (further addressed below). Nonetheless, the observations of an occluded DNA-binding site and the coulombic colored surface of the catalytically dead polymerase invite the prediction that C-Pol2 might have lost the ability to bind to DNA. To address this, we performed gel-shift assays to probe the DNA-binding function of various (non-cross-linked) Pol epsilon variants. As expected, DNA binding was observed for the wild-type, full-length tetrameric complex, and for the isolated full-length Pol2 polypeptide, which contains the DNA synthesis domain^32^. The deltacat construct, lacking the catalytic domain but containing ancillary subunits Dpb3-Dpb4, also resulted in clear gel retardation, in line with the notion that these histone-like proteins bind to DNA^31^. Importantly, no DNA binding was detected for C-Pol2, Dpb2 or a complex of the two (Fig. 3d and Supplementary Fig. 3). This observation is in line with the occluded DNA-binding site observed from the structure and indicates that the essential modules in the eukaryotic leading-strand polymerase are not DNA-binding factors.

### Structure of CMG-Pol epsilon on a pre-formed DNA fork

**Fig. 4:**
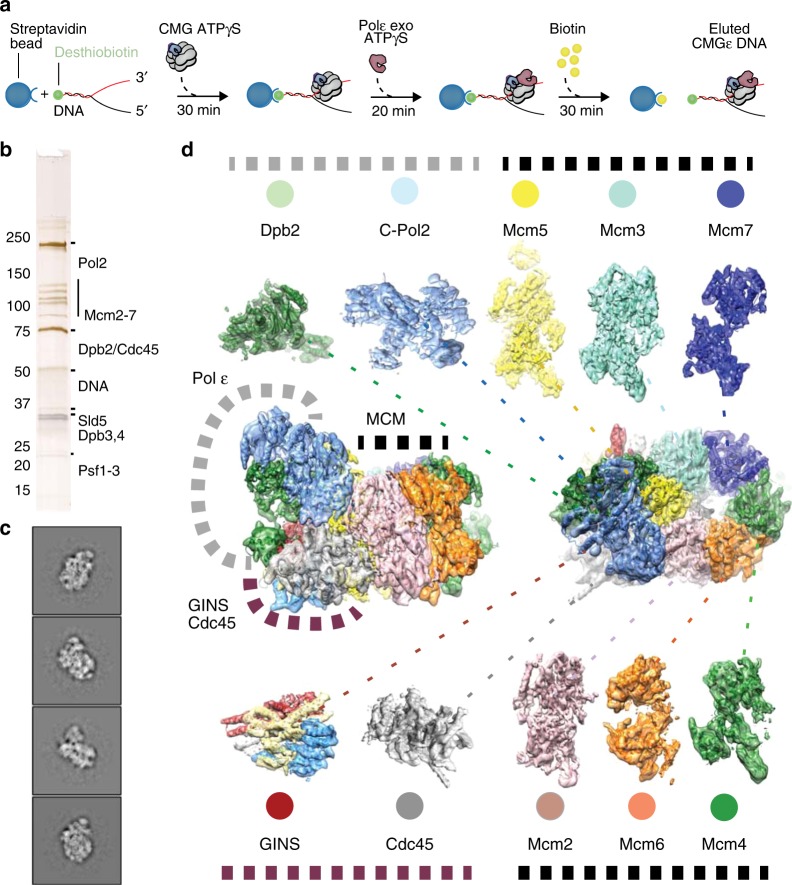
Cryo-EM structure of CMG-Pol epsilon. **a** Diagram of the reconstitution of CMG-Pol epsilon on a pre-formed fork. CMG stands for Cdc45-MCM-GINS. **b** Silver-stained SDS-PAGE gel of the reconstituted CMG-Pol epsilon-DNA complex. **c** Two-dimensional class averages of the CMG-Pol epsilon-DNA complex. **d** Cryo-EM structure of CMG-Pol epsilon with docked/real-space refined homology models of CMG and C-Pol2/Dpb2 components

**Fig. 5:**
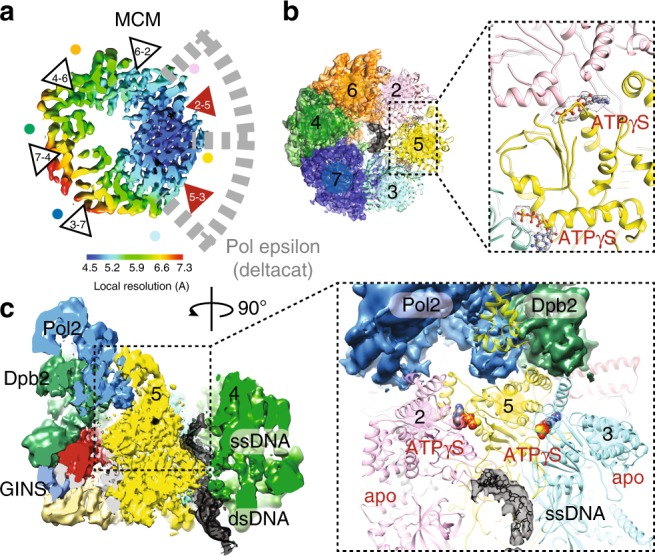
ATPase state and DNA binding in the CMG-Pol epsilon complex. **a** Inspection of the local resolution map indicates that the ATPase site clamped by C-Pol2/Dpb2 is highly stable, with the Mcm5 AAA+ module reaching resolutions as high as 4.5 Å. **b** Single-stranded DNA is captured by ATPase pore loops of Mcm3, -5, -2, and -6. ATPγS is bound to the Mcm5-3 and Mcm2-5 subunits. **c** A cut-through view of the CMG-Pol epsilon reveals duplex DNA entering through the N-terminal domain of MCM and the leading-strand template captured within the ATPase domain. A 90° rotated view of the Mcm3-5-2 region reveals how C-Pol2/Dpb2 clamping stabilizes nucleotide binding and single-stranded DNA engagement by Mcm2, Mcm5 and Mcm3 pore loops

To understand the function of the essential C-Pol2 and Dpb2 modules during replisome progression, we determined the structure of Pol epsilon associated with a fork-engaged CMG complex. To this end, we produced yeast CMG using a single yeast overexpression strain^28^. We added a slowly hydrolysable nucleotide analog (ATPγS) to stabilize DNA binding by the MCM motor^23^. Using these reagents, we immobilized the CMG on streptavidin-coated magnetic beads, bearing a desthiobiotin-labeled pre-formed DNA fork as bait. A full-length Pol epsilon variant containing inactivating mutations in the N-terminal Pol2 exonuclease was used to prevent DNA-fork degradation. Because desthiobiotin has reduced affinity for streptavidin compared to biotin, elution with biotin was highly efficient, yielding a nucleoprotein preparation suitable for single-particle cryo-EM analysis (Fig. 4a, b and Supplementary Fig. 4). To maximize particle numbers, we have absorbed our specimen using two subsequent applications, on lacey grids coated with an additional layer of ultrathin carbon. Particles were imaged on a Titan Krios EM with a K2 detector in counting mode. High-resolution 2D averages showed recognizable CMG assemblies with Pol epsilon decorating the ATPase tier of MCM, as previously reported in low-resolution negative-stain studies^21,28^ (Fig. 4c). Although end-on views of the MCM ring constituted the majority of particles, side and tilted views were sufficiently represented to yield an isotropic structure (Supplementary Fig. 5). Three-dimensional reconstruction based on 78,556 particles yielded a structure with an average resolution of 4.9 Å and local resolution estimation revealed marked variations, ranging from 4.5 Å in the ATPase core of MCM to 7.5 Å on the Pol2 surface (Fig. 4d and Supplementary Fig. 5-6). This resolution is easily sufficient for a reliable docking of yeast CMG^44^ and of the new atomic model of Pol epsilon deltacat (this study), both of which were built on higher-resolution cryo-EM maps. Whereas docking of yeast CMG required fitting of individual subdomains of the N-terminal and ATPase tiers as independent rigid bodies^25,26,44^, deltacat could be docked into unoccupied density as one rigid body, resulting in an unambiguous fit (Fig. 4d and Supplementary Movie 3). Our observation indicates that deltacat maintains the same compacted configuration even when crosslinking agents are omitted from the preparation, when full-length Pol epsilon is used and when the polymerase is bound to CMG. Notably, in the cryo-EM map, the catalytic domain of Pol epsilon is invisible, as previously reported for non-crosslinked CMG-Pol epsilon preparations^21,28^. Our structure shows that the CMG is stably interacting with the duplex/single-stranded DNA junction of the replication fork. Here, the double helix enters the MCM pore through the N-terminal tier, and single-stranded DNA (the leading-strand template) is captured by a set of ATPase pore loops (Mcm6-2-5-3, Fig. 5). As preported in previous CMG-DNA-fork structures, the lagging strand template, excluded from the MCM ring, cannot be resolved in our structure^44^. Furthermore, no obvious density for single-stranded DNA could be recognized bound to Pol2/Dpb2, confirming the notion that the essential catalytically inactive modules of Pol epsilon do not bind to DNA.

The observation of MCM interacting with the double helix in our ATPγS-CMG-Pol epsilon complex is noteworthy, given that previous studies on the CMG bound to a non-hydrolysable ATP analog showed single-stranded DNA inside the ATPase ring but no visible duplex DNA^25,44^. Productive engagement of the DNA duplex was instead previously captured only for a subset of translocating particles, in an ATP-CMG-fork preparation, halted by a DNA roadblock placed on duplex DNA^44^. To explain the difference in CMG-fork nexus binding in the presence or absence of the polymerase, we hypothesized that Pol epsilon association with the ATPase tier of ATPγS-CMG-DNA might cause a conformational change that promotes interaction with the DNA junction. Close inspection of the ATPase structure in ATPγS-CMG-Pol epsilon-DNA indicates that polymerase association indeed alters the configuration of MCM active sites, causing compaction of the Mcm3-5-2 subunits (with the highest local resolution in the cryo-EM map centered around Mcm5, Fig. 5a) and relaxation of the neighboring ATPase interfaces. Concomitantly, the nucleotide occupancy state in the ATP hydrolysis centers changes, with density peaks compatible with ATPγS detected in the Mcm5-3 and Mcm2–5 ATPase sites, while all other sites appeared nucleotide-free (Fig. 5b). This occupancy pattern differs from that of ATPγS-CMG-DNA or ATP-CMG-DNA-roadblock, where the Mcm6-2 interface is nucleotide-engaged, alongside Mcm5-3 and Mcm2-5^44^. This change in the ATPase structure likely alters DNA engagement and might be the reason why fork nexus binding can be observed in our ATPγS-bound CMG-Pol epsilon structure (Fig. 5c). Morphing between the ATP-CMG-DNA-roadblock and ATPγS-CMG-DNA-Pol epsilon structures show structural changes in the two states appear to promote a slight rotation of the double helix as DNA advances towards the MCM central channel (Supplementary Movie 4). This observation starts to provide insights into how changes in the ATPase state might promote helicase movement along the DNA^23,45–47^.

### Analysis of CMG-Pol epsilon contacts

**Fig. 6:**
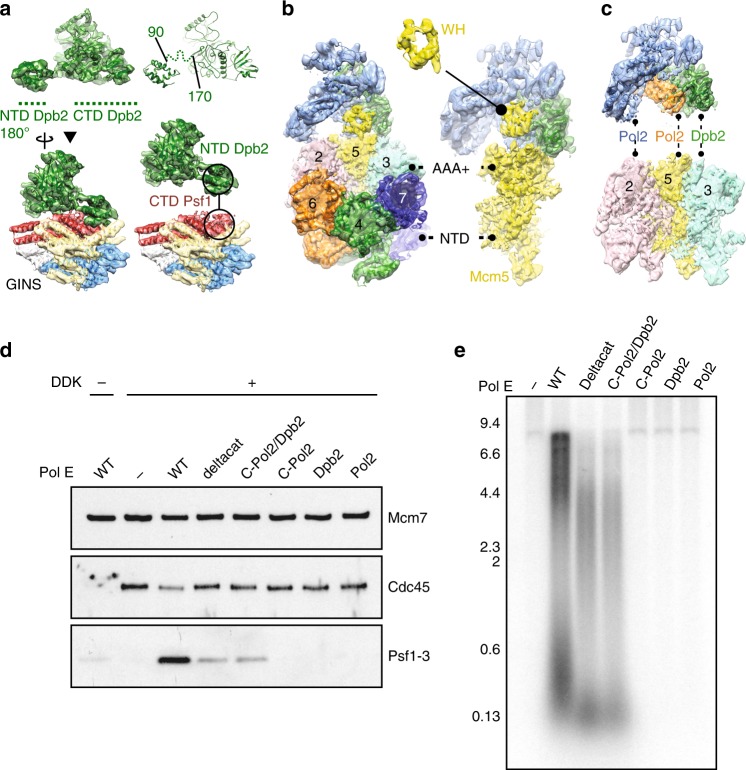
Interactions between Pol epsilon and the CMG. **a** A model for full-length Dpb2. N-terminal and C-terminal domains are tethered by a flexible linker. N-terminal Dpb2 contacts the C-terminal domain of GINS subunit Psf1. **b** The winged helix domain of Mcm5 is clamped between the catalytically dead polymerase in Pol2 and the zinc-finger appendix. **c** C-Pol2 and Dpb2 keep the ATPase domains of Mcm2-5-3 in a compacted state. C-Pol2 interacts with Mcm2 (via the polymerase fold) and Mcm5 (via the ZF2 element in the zinc-finger appendix, also see Fig. 2c). Dpb2 contacts the Mcm3 ATPase domain. **d** A complex containing both C-Pol2 and Dpb2 represents the minimal complement of Pol epsilon modules required for assembly of the CMG. **e** A complex of C-Pol2/Dpb2 but not the two isolated protomers support DNA replication in vitro

Close inspection of the cryo-EM map after docking of the CMG and deltacat structures highlighted unoccupied density. One region departs from the N-terminus of CTD-Dpb2 and projects as an extended arm towards GINS (Fig. 6a). Although the local resolution in this region of the map is relatively low (i.e., 6.5–7.5 Å resolution, as seen in Supplementary Fig. 5), an accurate fit can be obtained from docking of N-terminal Dpb2, generated by homology modeling based on the nuclear magnetic resonance structure of the human ortholog^37^. The resolution in this region is not sufficient to see any of the amino acid side chains involved in the GINS contact; however, inspection of our docking result provides confidence in the overall architecture of our model, given that the C-terminus of NTD-Dpb2 points towards the N-terminus of the CTD-Dpb2 module described above (Fig. 6a). The two domains appear tethered by a flexible linker, explaining why NTD-Dpb2 becomes visible only when stabilized by partner–protein interactions. In fact, inspection of the CMG contacts reveals that N-terminal Dpb2 engages C-terminal Psf1, in agreement with biochemical studies on Pol epsilon replisome incorporation by the Labib group (Fig. 6a)^30^. A second contact point involves the C-terminal winged helix (WH) appendix of Mcm5, which is clamped between the inactive polymerase and zinc-finger modules of Pol2 (Fig. 6c). All docked structures were subjected to real-space refinement using a combination of Coot^48^ and Phenix^49^.

Notably, in previous MCM-containing structures, the Mcm5 WH domain can be found occluding the MCM central channel when the ATPase motor is not DNA engaged^26,50,51^. We speculate that, by pulling the Mcm5 WH domain away from the central channel, Pol2-Dpb2 might promote productive DNA engagement inside the MCM pore. This is in agreement with the observation that ATPγS-CMG-Pol epsilon intimately engages the double helix at the DNA fork, as described above (Fig. 5c). Other CMG-Pol epsilon contacts can be visualized between C-Pol2/Dpb2 and the MCM AAA+ tier. For example, Pol2 keeps the Mcm2-5 ATPase gate shut, with the dead polymerase module touching Mcm5 and the zinc-finger appendix touching Mcm2. In particular, the ZnF2 element mapping in the Pol2 C-terminus contacts the Mcm2 ATPase, explaining a temperature-sensitive phenotype for cysteine-to-alanine substitutions in this peripheral element^35^. Likewise, the Dpb2 core sits across the Mcm5-3 ATPase interface (Fig. 6c). A structural role of Pol2 and Dpb2, clamping Mcm2-5-3 ATPase domains close together, agrees with our observation that the active sites at the Mcm2-5 and Mcm5-3 interfaces are ATPγS occupied, while other ATPase sites in the MCM ring are empty (Fig. 5a–c).

We note that the role of the C-Pol2/Dpb2 elements contacting MCM appears to match the function of GINS/Cdc45 in the CMG, although the two pairs of factors act on opposed tiers of the helicase ring^24–26^. In fact, GINS/Cdc45 latch across the Mcm2-5 gate and stabilize the Mcm5-3 interface by engaging the N-terminal collar^24–26^, while C-Pol2/Dpb2 are poised to stabilize the Mcm2-5/5-3 AAA+ interfaces (i.e., the sites essential for DNA unwinding^23^). Our new helicase–polymerase structure contains the pre-formed CMG bound to Pol epsilon on a model fork substrate and informs us on the architecture of the activated helicase poised to unwind the replication fork. We postulate that interactions seen in our structure might be also important for replication initiation. Two notions are key in this context. First, GINS and Pol epsilon are known to be recruited onto the MCM during the same step towards origin activation^19^. Second, stable CMG formation requires release of ADP and binding of ATP^2^. Our finding that Pol2 and Dpb2 directly contact three key ATPase domains of MCM suggests a mechanism by which this nucleotide exchange might be modulated.

### The role of C-Pol2 and Dpb2 in CMG formation

Our structure of deltacat Pol epsilon reveals an extensive interaction interface between C-Pol2 and Dpb2 (Fig. 2a). Furthermore, our structure of the CMG-Pol epsilon complex, reconstituted on the fork using recombinant active helicase and polymerase, establishes that Dpb2 mainly interacts with GINS (Fig. 6a) and C-Pol2 mainly contacts the MCMs (Fig. 6b). Given these observations, we asked whether elements of our CMG-Pol epsilon structure on the DNA fork would be retained during the process of Pol epsilon-dependent CMG assembly, on path to origin firing. We postulated that GINS recruitment and CMG formation might only occur when both Dpb2 and C-Pol2 are present in the same complex, as the physical link between GINS and MCM would be preserved. To test this hypothesis, we used a yeast replication system reconstituted with purified proteins^13^ to establish the minimal complement of Pol epsilon domains that are required to make stable CMG. To this end, we loaded MCM double hexamers onto an immobilized DNA fragment and added a full complement of firing factors to promote the assembly of a stable CMG complex^2,13^. As shown in Fig. 6d, when wild-type Pol epsilon was substituted for deltacat, CMG was still assembled, although less efficiently. A Pol epsilon variant containing both C-Pol2 and Dpb2 showed the same efficiency of CMG formation as deltacat, indicating that ancillary subunits Dpb3/Dpb4 are not required for CMG assembly. As we predicted, the isolated full-length Pol2, C-Pol2, or Dpb2 subunits were unable to support CMG formation (Fig. 6d). We then tested whether the CMG assembled by these Pol epsilon variants could support DNA replication, by additionally including the proteins required for complete leading and lagging-strand synthesis^52^. Only wild-type Pol epsilon, deltacat, and C-Pol2/Dpb2 supported DNA replication (Fig. 6e). As previously reported, DNA replication products in the presence of deltacat were shorter compared to wild-type Pol epsilon, consistent with the idea that in the absence of the Pol2 catalytic domain, leading-strand synthesis by *trans*-acting Pol delta occurs at a slower rate^52^. Importantly, in reactions containing C-Pol2/Dpb2, we observed the same amount and profile of replication products as with deltacat. This in agreement with the notion that the remaining Dpb3 and Dpb4 subunits do not play a direct role in the DNA replication reaction, but rather modulate histone redeposition at the replication fork^53,54^. As expected from their defect in CMG assembly, the isolated Pol2, C-Pol2, and Dpb2 subunits failed to support DNA replication in this system (Fig. 6d–e). Altogether, our results are compatible with the idea that C-Pol2 and Dpb2 must act as a heterodimeric complex, to link GINS to the MCM during CMG establishment. Cryo-EM characterization of the complete CMG assembly reaction (including CDK, DDK, Sld2, Sld3/7, and Dpb11) will be key to understanding the essential structural role of C-Pol2/Dpb2 during replication initiation.

### Analysis of CMG productively bound to the replication fork

**Fig. 7:**
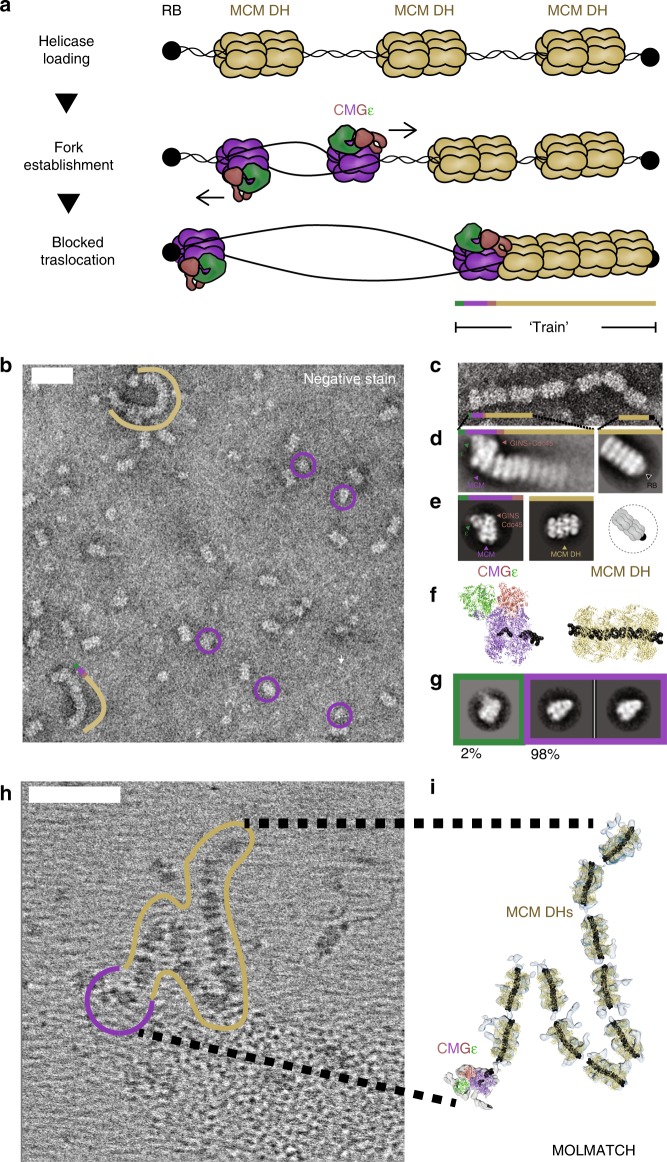
Composition of the replicative helicase productively engaged to the replication fork. **a** Diagram of the EM-based translocation assay. Multiple MCM double hexamers are loaded onto a linear stretch of duplex DNA capped with protein–DNA roadblocks. Only certain MCM double hexamers are activated and translocating CMGs push MCM double hexamers against the DNA roadblock. **b** Representative negative-stain micrograph reveals accumulation of MCM trains (marked with a khaki line) alongside isolated CMGs (marked with purple circles). Scale bar 50 nm. **c** Detail of a negative-stain micrograph revealing that MCM trains are made of stacked double hexamers loaded onto DNA. **d** Two-dimensional averages of the CMG capping the trains and opposed end of trains capped by a protein roadblock. Most MCM-pushing CMG particles are bound to Pol epsilon. **e** 2D averages of CMG-Pol epsilon and MCM double hexamers, next to a cartoon of an MCM blocked by a protein–DNA roadblock. **f** Atomic structures of CMG-Pol epsilon and MCM double hexamer, both bound to DNA. **g** 2D class averages of isolated CMGs observed in the MCM train experiment. Most CMGs that failed to translocate up to the roadblock are not engaged by Pol epsilon. **h** Cryo-electron tomogram of an MCM train. Scale bar 50 nm. **i** MCM and CMG structures placed into the cryo-electron tomogram using template matching approaches. This experiment supports the notion that CMG-Pol epsilon particles push MCM double hexamers against a protein–DNA roadblock to form MCM trains

During replisome progression, it is unknown whether C-Pol2 and Dpb2 indeed remain anchored to the ATPase domain of the advancing CMG. To address this issue, we reconstituted CMG formation and Mcm10-dependent DNA unwinding using the in vitro system with purified yeast proteins^13^. Briefly, origin activation reactions were assembled on linear, biotinylated DNA tethered to streptavidin-coated magnetic beads. The linear DNA fragment was blocked at the two ends with covalent protein–DNA roadblocks to prevent linear diffusion of MCM off the DNA. This substrate was used to load multiple MCM double hexamers and firing factors were added to promote origin activation. Subsequently, elution from the streptavidin beads was achieved by DNA digestion at a single site, leaving the doubly blocked DNA segment intact. Using 2D classification of negatively stained data, we observed that only a subset of these double hexamers were converted to CMG, causing inactive MCM double hexamers to be pushed along duplex DNA in front of the helicase and against the protein–DNA roadblock^2^ (Fig. 7a). This resulted in the formation of stacks of MCM particles (trains), which are capped at one end by a CMG engine (Fig. 7b–d). We can exclude the possibility that trains are the mere product of protein aggregation, because they disappear when covalent protein roadblocks are omitted, or when the Mcm10 firing factor is excluded from the origin activation reaction (Supplementary Fig. 7)^2^. To define the identity of the helicase productively engaged to the DNA fork, we first analyzed MCM-containing particles that were not train incorporated. These particles fell in two categories: isolated MCM double hexamers and CMGs (Fig. 7e–g). Remarkably, isolated CMGs were largely polymerase free, with only a small fraction (2% of recognizable, averaged particles) of the helicase bound by Pol epsilon (Fig. 7g). To establish the composition of the active CMG productively bound to the DNA fork, we then focused our analysis on the tips of the MCM trains. We confirmed that one end of the train was capped by the protein roadblock found in direct contact with an MCM double hexamer (Fig. 7e). At the opposed end of the train, the vast majority of CMGs (75–100% of recognizable, averaged particles in different repeats of the same experiment) were bound by a AAA+ interacting Pol epsilon complex (Fig. 7d). Our observation was further supported by cryo-electron tomographic analysis (Fig. 7h). In this experiment, we used a template matching approach (as implemented in MOLMATCH^55^) to recognize MCM double hexamers and CMG particles in the MCM trains. As predicted from our negative-stain experiment, we found high correlation peaks for MCM double hexamers along the length of the train and one CMG particle capping one end of the train. We also observed that a Pol epsilon-bound form of CMG best matches the cryo-tomographic density in this region (Fig. 7i, Supplementary Fig. 7, and Supplementary Movie 5).

In summary, these data indicate that the translocating form of the CMG is Pol epsilon bound. Combined with our cryo-EM reconstruction of the fork-bound CMG-Pol epsilon, our results suggest that tight Pol epsilon binding to the AAA+ tier of the MCM ring might stabilize productive helicase engagement to the duplex/single-stranded DNA junction.

## Discussion

In this study, we have provided the first structural evidence that the catalytic subunit Pol2 contains a tandem repeat of polymerase modules^33^ (Figs. 1a, 2a–c). In Pol2, the N-terminal repeat contains the DNA synthesis function^32^, while the C-terminal repeat is catalytically inactive^35^. We showed that C-Pol2 shares an extended interface with the Pol epsilon subunit Dpb2, which in turn contains a catalytically dead exonuclease^34^ (Figs. 1d, 2a,b). Having evolved from two DNA-processing enzymes, not only have C-Pol2 and Dpb2 lost their catalytic activity^43^ but also their DNA-binding functions. In fact, we found that only Pol epsilon permutations that contain the N-Pol2 catalytic module^32^ or histone-like factors Dpb3-Dpb4^31^ can bind to DNA, as observed in gel-shift assays. Conversely, no DNA association could be observed for C-Pol2 or Dpb2 (Fig. 3d).

Our cryo-EM structure of an isolated Pol epsilon deltacat derivative explains why C-Pol2 cannot bind to DNA. In particular, the inner surface in the polymerase fold is not as positively charged as the DNA-binding groove of other replicative polymerases such as Pol alpha^41,43^, and it is further occluded by the C-terminal zinc-finger appendix of Pol2 (Fig. 3a–c). In line with these observations, our cryo-EM structure of the DNA-engaged CMG-Pol epsilon showed DNA binding by MCM proteins, but not by C-Pol2 and Dpb2 (Fig. 5c; while, due to flexibility, N-Pol2 and Dpb3-Dpb4 were not visible). Given that these are not enzymes nor DNA-binding factors, we reasoned that C-Pol2 and Dpb2 must play a structural role in the replisome. This scenario is reminiscent of Cdc45 that has evolved from a RecJ exonuclease^56^. Cdc45 has lost its DNA-processing functions, while it has acquired a key structural role in reconfiguring the MCM motor, to promote DNA melting and fork unwinding within the CMG^23,24^.

**Fig. 8:**
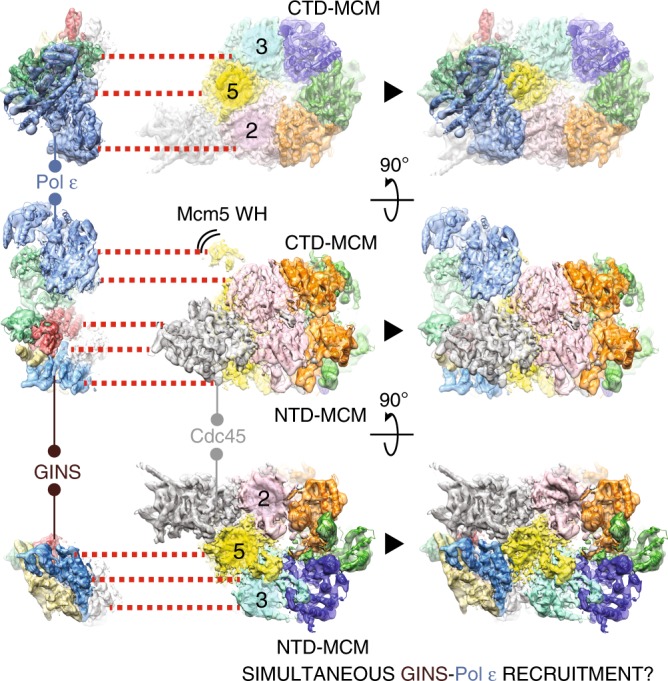
Proposed mechanism for the concomitant recruitment of GINS and Pol epsilon onto MCM. By interacting with Cdc45 on the N-terminal MCM face, GINS stabilizes the Mcm2-5-3 N-terminal interactions. C-Pol2 and Dpb2 in the Pol epsilon complex play a similar role, by stabilizing the Mcm2-5-3 AAA+ ATPase interactions. Given that GINS and Pol epsilon are recruited onto MCM as part of the same complex, concomitant biding of both factors might promote activating conformational changes in the helicase, eventually leading to origin activation

In support of a structural role for Dpb2 and C-Pol2, inspection of the DNA fork-CMG-Pol epsilon structure reveals that these two modules provide a molecular bridge across CMG components, with Dpb2 mainly contacting GINS, and C-Pol2 contacting MCM (Fig. 6a–b). GINS and Pol epsilon are recruited onto MCM-Cdc45 as part of the same pre-loading complex^19^. Previous work on the CMG structure established that GINS and Cdc45 bind to the N-terminal tier of Mcm2-5-3, stabilizing a planar configuration of the MCM ring and locking a natural gate, between MCM subunits 2 and 5^24–26,57,58^. In the CMG-Pol epsilon structure, we now found that C-Pol2/Dpb2 interact with the same Mcm2-5-3 protomers, poised to stabilize the C-terminal AAA+ interfaces. Our observation is noteworthy, given that Mcm2-5 and Mcm5-3 contain the two ATPase centers that are essential for CMG function^23^, and suggests a mechanism for the simultaneous recruitment of GINS and Pol epsilon (Fig. 8).

A direct contact between GINS/Pol epsilon and MCM might play a role both during origin activation as well as replication fork advancement. To test the idea of a role during activation, we have used in vitro reconstitution of DNA replication^2,13^, to show that a combination of C-Pol2 and Dpb2, but neither of the two factors in isolation, constitute the minimal complement of Pol epsilon modules required for CMG formation (Fig. 6d–e). Therefore, key aspects of the CMG-Pol epsilon architecture described in our cryo-EM map appear to be important for origin activation^35,43^.

Our structural studies also provide insights into replication fork progression. In comparing our new DNA-CMG-Pol epsilon structure to the published DNA-CMG structure (PDB entry 5U8S)^44^, we found that C-Pol2/Dpb2 association with the C-terminal face of MCM alters the ATPase conformation by stabilizing the Mcm5-ATPγS-Mcm3 and Mcm2-ATPγS-Mcm5 interactions, while all other active sites are nucleotide-free (Fig. 5a–b). In these conditions, and for the first time in an ATPγS-CMG complex, the duplex/single-stranded DNA junction, and not just single-stranded DNA, can be seen engaged by the MCM complex. This feature is shared by all particles that contribute to high-resolution 3D structures in our dataset (Fig. 5c). Strikingly, fork nexus engagement was previously only observed when the helicase was incubated with ATP to promote DNA translocation and only in a small subset of helicase particles^44^. Together with our EM observation that the translocating form of the CMG is Pol epsilon bound (Fig. 7), our data support the notion that polymerase binding promotes productive DNA fork engagement by the CMG.

Furthermore, our structure informs a model for helicase translocation at the replication fork. Previous structural reports on the *Drosophila* and yeast CMG helicase, from us^25^, the Berger/Botchan^24^, and from the O’Donnell/Li^26^ laboratories, showed that the Mcm2-5 interface is highly dynamic and that ATP binding can shift the equilibrium from a relaxed (nucleotide-free) to a tight (nucleotide-bound) state of the ATPase side of the MCM ring in the CMG. This observation led to us and others suggest that DNA translocation could occur with the helicase “inchworming” along DNA through relaxing and tightening of the Mcm2-5 AAA+ interface (referred to as “pump-jack model” in the yeast study)^25,26^. Alternatively, DNA translocation would occur via a distinct mechanism that still needs to be determined, while the relaxed Mcm2-5 AAA+ structure in the CMG could represent a pausing state of the helicase^25^. Our new structural data appear to support the latter model, as Pol epsilon engagement by the CMG seems incompatible with the conformational rearrangements described in the pump-jack helicase^8,26^. The three structural changes described in the pump-jack model involve opening of a gap between Mcm2 and Mcm5, transition from a planar to a spiral ATPase tier and movement of the Mcm5 WH domain. These three conformational switches would seemingly all contribute to disrupting the multipartite Pol epsilon-binding site on the CMG (Supplementary Movie 6). In this study, we also provide evidence for Pol epsilon association occurring when CMG particles are productively fork engaged. This is why we now favor a model whereby a wide Mcm2-5 ATPase opening, which is incompatible with Pol epsilon binding, would cause helicase stalling (proposed before by Botchan and Berger^59^), not DNA translocation. Alternatively, engagement between CMG and deltacat could be much more dynamic than we would predict by inspecting our structure. Future studies are needed to understand the relation between ATP binding/hydrolysis, helicase movement, and replicative polymerase exchange rates in the advancing replisome. The key to understanding replisome progression, we predict, will likely be fork stabilization factors Mrc1/Csm3/Tof1, which all contribute to achieving cellular rates in DNA replication reactions reconstituted in vitro^52^.

## Methods

### Yeast expression strains

Strains are based on W303. See Supplementary Table 1 for a list of all strains and genotypes used in this study.

### Protein expression and purification

For expression of wild-type Pol epsilon and dropout variants, cells were grown in YEP media supplemented with 2% raffinose. Cells were arrested in the G1 phase at a density of ~2–3 × 10^7^ cells/ml with 100 ng/ml alpha factor for 3 h at 30 °C. Protein expression was then induced by adding galactose to 2% and growth continued for 3 h at 30 °C. Cells were harvested by centrifugation at 4000 rpm for 30 min in a Beckman Coulter J6-MC Centrifuge (Beckman JS-4.2 rotor) and washed with Buffer E (25 mM HEPES, pH 7.6, 10% glycerol, 1 mM dithiothreitol (DTT)) supplemented with 500 mM KOAc (Buffer E-500). Cells were spun down again in a Beckman Coulter Allegra^®^ X-15R Centrifuge and resuspended with Buffer E-500 (at half the pellet volume) and frozen dropwise into liquid nitrogen. Frozen droplets were crushed in a 6875D Freezer/Mill^®^ Dual Chamber Cryogenic Grinderfreezer mill (SPEX SamplePrep) using six cycles, intensity 15 (Precool 1 min, run 2 min, break 1 min), operating at −80 °C. Cell powder was resuspended in Buffer E supplemented with complete protease inhibitor tablets (Roche).

Pol epsilon and dropout variants were prepared as follows: the powder was resuspended with 250 ml Buffer E supplemented with 400 mM KOAc (Buffer E-400) and protease inhibitors (Roche). Lysate was cleared by ultracentrifugation at 45,000 rpm for 1 h at 4 °C (Ti45 rotor, Beckman Coulter Optima L-100 XP Ultracentrifuge). For all proteins expressing a CBP tag (wild type, exo^−^, and deltacat), 3 ml calmodulin beads (GE Healthcare) were pre-equilibrated in Buffer E-400, added to the cleared lysate, and supplemented with 2 mM CaCl_2_. For the remaining proteins (C-Pol2, C-Pol2/Dpb2, Dpb2, Pol2), 4 ml anti-Flag M2 affinity resin was pre-equilibrated in Buffer E-400 and added to the cleared lysate. All incubations occurred at 4 °C with end over rotation for 2 h. Flow-through was collected and beads were washed with 100 column volumes of Buffer E-400 (supplemented with 2 mM CaCl_2_ for the CBP-tagged proteins). Proteins were eluted either using elution Buffer E-400 supplemented with 2 mM EGTA and 2 mM EDTA (CBP tagged proteins) or with Buffer E-400 supplemented with 1 mg/ml FLAG peptide (FLAG-tagged proteins). The elutions were pooled and injected into SP Sepharose Fast Flow 1 ml column (GE Healthcare) attached to a Mono Q 5/50 GL 1 ml column (GE Healthcare) in an Äkta purifier (GE Healthcare). Proteins were washed with either 20 CV Buffer E-300 (for all variants except exo^−^) or Buffer E-400 (exo^−^). Proteins were eluted after removal of SP Sepharose Fast Flow column over a 15 CV gradient (Buffer E 300–1500 mM KOAc for all variants apart from exo^−^, where a 400–1000 mM KOAc gradient was used). The purest fractions were pooled either dialyzed or buffer exchanged to Buffer E-400 via gel filtration using a Superdex 200 Increase 3.2/300 column for cryo-EM or Superdex 200 10/300 GL for DNA-binding assays.

CMG purification was performed as follows: yJCZ3 was used to express CMG^28^. Induction was performed using 2% galactose for 3 h at 30 °C, frozen dropwise in liquid nitrogen, and ground as decribed above. Cell powder was resuspended in buffer C (25 mM HEPES, pH 7.6, 0.02% Tween-20, 10% glycerol, 1 mM EDTA, 1 mM EGTA) supplemented with 15 mM KCl, 2 mM MgCl_2_, 2 mM β-mercaptoethanol, and complete protease inhibitors mixture. KCl was then added to 100 mM, and the lysate cleared by centrifugation. The clear lysate was incubated with anti-Flag M2 affinity resin at 4 °C, washed with C-100 buffer (buffer C with 100 mM KCl and 1 mM DTT), and eluted with C-100 buffer supplemented with 0.5 mg/ml FLAG peptide and complete protease inhibitors mixture. Peak fractions were pooled and loaded onto a HiTrap SP FF (GE Healthcare). The flow-through was collected and loaded onto a Mono Q 5/50 GL (GE Healthcare), washed with C-100 buffer, and eluted over 100 mM to 550 mM KCl gradient in C-100 buffer supplemented with 1 mM DTT. Peak fractions were pooled, diluted to 150 mM KCl in buffer C, loaded onto Mono Q 1.6/5 PC in buffer D (25 mM HEPES, pH 7.6, 1 mM EDTA, 1 mM EGTA, 1 mM DTT) supplemented with 150 mM KCl, and eluted over 150 mM to 550 mM KCl gradient in buffer D.

### DNA sequences

All oligonucleotides were purchased from integrated DNA technologies (IDT). Oligonucleotides used for electrophoretic mobility shift assays were:

160 nt forward,

5′-ACCGATGTGGTAGGAAGTGAGAATTGGAGAGTGTGTTTTTTTTTTTTTTTTTTTTTTTTTTTTTTTTTTTTTTTTGAGGAAAGAATGTTGGTGAGGGTTGGGAAGTGGAAGGATGGGCTCGAGAGGTTTTTTTTTTTTTTTTTTTTTTTTTTTTTTTTTT-3′

37 nt reverse,

5′-CCACTCCCAACCCTTCACCTTCCTACCCGAGCTCTCC-3′.

Oligonucleotides used for fork DNA affinity purification of CMG-Pol epsilon were:

leading-strand template,

5′/5deSBioTEG/GCAGCCacgctGGCCGTTTTACAACGTCGTGACTGGGCACTTGATCGGCCAACCTTTTTTTTTTTTTTTTTTTTTTTTTTTTTTTTTTTTTTTT-3′

lagging-strand template,

5′-GGCAGGCAGGCAGGCAGGCAGGCCGTGCGCGTGGTCGTGCGGTTGGCCGATCAAGTGCCCAGTCACGACGTTGTAAAACGGCCAGCGTGGCTGC-3′

To anneal the DNA, the two oligonucleotides were mixed in an equimolar ratio and left to incubate at 95 °C for 3 min followed by slow cooling to room temperature.

### Electrophoretic mobility shift assays

Purified wild-type Pol epsilon and dropout variants were pre-mixed in a dilution series (75, 150, 200, 300, and 450 nM) with DNA at a concentration of 300 nM, in a reaction buffer containing 25 mM HEPES, pH 8.0, 30% glycerol, 2 mM EDTA, 0.2 mg/ml bovine serum albumin (BSA), and 0.02% Triton-X. Binding was performed for 30 min at 4 °C. polyacrylamide *gel* electrophoresis (PAGE) made with 4% polyacrylamide, 3% glycerol, and 0.5× TAE polymerase was pre-run at 100 V for 1 h (4 °C). Protein–DNA complexes were resolved by running the PAGE at 100 V for ∼2 h (4 °C) in 0.5× TAE. Visualization of the complexes were done my staining the PAGE with Diamond Nucleic acid dye (Promega) for 15 min. Imaging was done with 1.5 s manual exposure using Bio-Rad Gel DOC XR+ equipped with the Bio-Rad image lab software.

### Reconstitution of CMG Pol epsilon bound to a pre-formed fork

To reconstitute CMG-Pol epsilon on DNA, a bead affinity approach was used. Seven microliters of M-280 Streptavidin Dynabeads™(Thermo Fisher) slurry was added to low-binding microcentrifuge tubes and all incubations occurred at 30 °C with 1250 rpm shaking in a thermomixer. Beads were washed twice with 20 µl DNA-binding buffer (25 mM HEPES 7.6, 1 M NaCl, 10% glycerol, 0.01% NP-40, 1 mM EDTA). Twenty microliters of 250 nM fork DNA was added and then incubated for 30 min. The supernatant was collected and the beads were washed once with 20 µl DNA-binding buffer and once with 20 µl protein-binding buffer (25 mM HEPES 7.6, 100 mM KOAc, 10 mM Mg(OAc)_2_, 10% glycerol, 0.01% NP-40, 1 mM DTT, 2 mM ATPγS). Twenty microliters of 250 nM CMG was added and the reaction was incubated for 30 min. The supernatant containing unbound CMG was collected and beads were washed once with 20 µl protein-binding buffer. Twenty microliters of 80 nM polymerase epsilon exo^−^ was added to the beads and then incubated for 20 min at 30 °C and 1250 rpm. Beads were washed once with 20 µl protein-binding buffer and once with 20 µl protein-binding buffer without glycerol. Complexes were eluted by the addition of 10 µl elution buffer buffer (25 mM HEPES 7.6, 100 mM KOAc, 10 mM Mg(OAc)_2_, 0.01% NP-40, 1 mM DTT, 2 mM ATPγS, 400 nM biotin) and then incubated for 30 min. Elutions were pooled and used for cryo-EM grid preparation.

### Cryo-EM grid preparation

Deltacat polymerase epsilon grids were prepared as follows: purified deltacat polymerase epsilon was first crosslinked with 0.01% glutaraldehyde and incubated for 5 min on ice prior to plunge freezing. Quantifoil R2/2 open-hole grids coated with a layer of freshly evaporated carbon (not covering holes) were glow discharged before applying 4-µl sample onto it. After a 30-s incubation, the grid was double-side blotted using a Vitrobot Mark IV (FEI Thermo Fisher), and then operated at 4 °C at 100% humidity, using a blotting time of 2.5 s.

CMG-polymerase epsilon exo^−^ was prepared as follows: freshly glow-discharged 400-mesh lacey grids containing an ultrathin layer of carbon (Agar Scientific) were used for vitrification in a Vitrobot Mark IV (Thermo Fisher) operating at 21 °C and 100% humidity. Samples were vitrified in a double application process. Four microliters of the sample was first applied to the carbon side of the grid and incubated for 120 s. The grid was then double-side blotted for 0.5 s before a further 4 µl sample was applied. After another 120 s incubation, the grid was blotted for 3 s and plunge frozen into liquid ethane.

### Data collection

Cryo-EM data for all samples were collected on a Titan Krios EM equipped with a Falcon III direct electron detector (FEI Thermo Fisher) or a K2 Summit direct electron detector (Gatan Inc.) at the Francis Crick Institute. See Supplementary Table 2 for image acquisition details.

### Image processing

Pre-processing of deltacat and CMG-polymerase epsilon exo^−^ were performed as follows: movie stacks were corrected for beam-induced motion and then integrated using MotionCor2^60^. All frames were retained and a patch alignment of 5 × 5 was used. Contrast transfer function (CTF) specifications for each non-dose-weighted micrograph were estimated by CTFFIND4^61^ and Gctf^62^. Good quality-integrated movies examined by Relion-2.1^63^ were selected for further image processing. Particle picking was performed in a semi-automated mode using e2boxer from EMAN2 (version 2.07)^64^. All further image processing was performed in Relion-2.1.

Deltacat particles were further treated as follows: processing of the Falcon III VPP data were performed as follows. Particle extraction was carried out from dose-weighted micrographs by setting the box size at 184 pixels (pixel size 1.09 Å/pixel. An initial model was generated using cryoSPARC^65^ and was used in processing with Relion-2.1^66^. An initial 816,814 particle dataset was cleaned by 2D classification followed by two subsequent rounds of 3D classification (performing alignment). 3D refinement yielded a 4.45 Å structure, which was filtered to the local resolution using LocRes in Relion-2.1 (Supplementary Table 2 and Supplementary Fig. 1 and 2).

CMG-Pol epsilon exo^−^ particles were further processed as follows: a first dataset with a total of 252,705 binned-by-2 particles were extracted from 8,782 dose-weighted micrographs by setting the box size at 256 pixels (pixel size 1.38 Å/pixel) and scaling down to 128 pixels (2.76 Å/pixel) from a first dataset. A second dataset with the same imaging parameters was collected, where a total of 151,971 binned-by-2 particles were extracted from 5,687 dose-weighted micrographs. The particles from the first dataset were subjected to 3D classification with alignment using the negative-stain CMG-polymerase epsilon structure (EMD-6465)^21^ as an initial model (filtered to 60 Å) for five classes. The “ignore CTFs until the first peak” option was selected at this stage and the mask size was set at 350 Å. The best two classes contained a total of 115,068 particles, which were re-extracted back to the unbinned parameters (256 pixels) and subjected to a cascade of 3D refinement and 3D classification without alignment (see Supplementary Fig. 6). The second dataset was subjected to rounds of 2D classification and the best particles (5671) were re-extracted to unbinned parameters (256 pixel box size) and combined with the 89,809 particles from the first dataset. The merged 95,480 particles were subjected to 3D classification with alignment (three classes) and the best two classes from that step were selected (78,556 particles) and subjected to homogeneous 3D refinement in cryoSPARC^65^. For post-processing, the LocRes function in Relion-2.1 was used by inputting the two unfiltered half maps from the cryoSPARC reconstruction and setting the *B*-factor to −300 Å.

### Molecular modeling

Homology models for yeast C-Pol2 and Dpb2 were generated using HHpred^40^, based on the atomic structure of yeast Pol1 (PDB entry 4FYD)^41^ and on the co-crystal structure of human Pole2/ZnF Pole1 (PDB entry 5VBN)^34^. Rigid-body docking was performed in UCSF Chimera^67^ and manually adjusted in Coot^48^ and subjected to real-space refinement in Phenix^49^. All fiigures were made using the UCSF chimera^67^.

### DNA replication reconstituted in vitro

CMG assembly assays were performed as in ref ^2^. Briefly, MCM was loaded onto 60 ng end-biotinylated DNA fragment containing ARS1 that was immobilized on M-280 streptavidin resin (Sigma) in a 10 µl reaction containing 37.5 nM ORC, 50 nM Cdc6, and 100 nM Mcm2–7:Cdt1. After 10 min at 30 °C, DDK was added to a final concentration 50 nM and the reaction incubated for a further 5 min. Buffer was added to a final concentration of 250 mM K-glutamate, 25 mM HEPES, 10 mM Mg-acetate, 0.02% NP-40-S, 8% glycerol, 400 μg/ml BSA, 5 mM ATP, and 1 mM DTT. A mix of firing factors was assembled immediately before use and added at time 0, to a final concentration of 50 nM Dpb11, 50 nM GINS complex, 50 nM Cdc45, 30 nM Pol epsilon, 20 nM Clb5–Cdc28 (CDK), 2.5 nM Mcm10, 30 nM Sld3–Sld7, and 55 nM Sld2 (firing factor mix). After 10 min at 30 °C, beads were washed twice with a buffer containing 25 mM HEPES 7.6, 5 mM Mg-acetate, 0.02% NP-40-S, 10% glycerol, and 250 mM K-glutamate, resuspended in sodium dodecyl sulfate (SDS)-loading buffer, separated by SDS-PAGE and analyzed by Western blotting with the antibodies indicated.

Replication assays were performed as in ref. ^52^, using an 8.2 kb origin-containing DNA template. After 40 min at 30 °C, replication reactions were quenched with EDTA and products separated on a 0.6% denaturing alkaline agarose gel.

### Negative-stain EM of the translocating replicative helicase

MCM trains were assembled by activating loaded MCMs on a capped linear DNA segment^2^. Negative-stain sample preparation was performed using carbon coated-300-mesh copper grids (EM resolutions). Grids were glow discharged for 30 s at 45 mA (Electron Microscopy Sciences). Three-microliter drops of nucleoprotein assemblies were applied to the grids and incubated for 1 min. Excess sample solution was blotted away and staining was performed on four separate 70-μl 2% uranyl acetate drops by stirring for 5, 10, 15, or 20 s. Excess stain was blotted away and grids were stored before imaging. Negative-stain micrographs were acquired on a Tecnai LaB6 G^2^ Spirit transmission electron microscope (FEI) operating at 120 keV. Micrographs were collected using a GATAN Ultrascan 100 camera at a nominal magnification of 21,000 (resulting in a pixel size of 4.92 Å), using a defocus range of −0.5 to 2.5 μm. Negative-stain particles were picked using EMAN2^64^, version 2.07, and further image processing was performed in Relion-2.1^66^. Particles were extracted with a box size of 128 × 128 pixels and subjected to reference-free 2D classification with the –only_flip_phases additional argument. Size of datasets was as follows: 405 micrographs (75,527 particles) were collected for the MCM train experiment; 228 micrographs (9302 particles) for the multi-site cut/minus methyltransferase control; 112 micrographs (7977 particles) for the single-site cut/minus methyltransferase control; 92 micrographs (4614 particles) for the single-site cut/minus methyltransferase/minus Mcm10 control; 82 micrographs (8013 particles) for the single-site cut/plus methyltransferase/minus Mcm10 control.

### Cryo-electron tomography

Twenty tilt series were collected in Tomography 4.0 on a Titan Krios (Thermo Fisher Scientific, Waltham, MA, USA) equipped with a GIF quantum energy filter with a slit width of 20 eV and K2 summit detector (Gatan, Pleasonton, CA, USA). Images were collected from ± 54° with a 3° increment at a pixel size of 2.71 Å. Each exposure received a dose of 3 e/Å^2^ that was fractionated across four movie frames for a total dose of 111 e/Å^2^. The defocus ranged from −5 to −7 μm. Movie frames were aligned with the alignframes function in IMOD and the tilt series was subsequently aligned using fiducial-less patch tracking option in IMOD. 2D CTF correction was performed with ctfphaseflip. Tomograms were reconstructed by weighted back-projection and subjected to a SIRT-like filter equivalent to 1 iteration.

Template matching of tomograms was performed with the MOLMATCH software^55^. Individual MCM and CMG maps filtered to 20 Å resolution were used as templates that were systematically rotated and translated before cross-correlation with a region of the tomogram. Euler angle range used for scanning was 0–360 for phi, 0–360 for psi, and 0–180 for theta, with a 10° increment. Missing wedge compensation was applied to the template, therefore constraining the cross-correlation to the experimentally sampled fraction of Fourier space. For each template, 3D coordinates and orientations that corresponded to the maximum correlation coefficients were extracted using the AV3 toolbox in Matlab (av3_createmotl)^68^. The top MCM and CMG matches were displayed against the tomogram with the EM Package for Amira 5.3^69^ (Thermo Fisher Scientific, Waltham, MA, USA), using the positions and orientations determined by MOLMATCH^55^.

## Electronic supplementary material

Supplementary Information

Description of Additional Supplementary Information

Supplementary Movie 1

Supplementary Movie 2

Supplementary Movie 3

Supplementary Movie 4

Supplementary Movie 5

Supplementary Movie 6

Reporting Summary

## Data Availability

Pol epsilon variant deltacat has been deposited with the EMBD with accession code EMD-0287 and the atomic model with the Protein Data Bank under accession code PDB 6HV8. CMG-Pol epsilon is deposited under EMDB entry EMD-0288 and the corresponding atomic model under accession code PDB 6HV9. A reporting summary for this Article is available as a Supplementary Information file. All experimental data are available upon reasonable request.
