# Supplementary material for: Structure of DNA-CMG-Pol epsilon elucidates the roles of the non-catalytic polymerase modules in the eukaryotic replisome
Source: Nat Commun. 2018 Nov 29;9:5061. doi: 10.1038/s41467-018-07417-1 (PMC6265327; doi:10.1038/s41467-018-07417-1)
Supplement: Supplementary file 1 — Supplementary Information [file 41467_2018_7417_MOESM1_ESM.pdf]

Supplementary material

Cryo-EM structure of DNA-CMG-Pol epsilon elucidates the roles of essential, non-catalytic polymerase modules in the eukaryotic replisome

Panchali Goswami[1,§], Ferdos Abid Ali[1,§], Max E. Douglas[2], Julia Locke[1],
Andrew Purkiss[3], Agnieszka Janska[2], Patrik Eickhoff[1], Anne Early[2], Andrea Nans[3],
Alan Cheung[4,5], John F. X. Diffley[2] and Alessandro Costa[1,*]

[1] Macromolecular Machines Laboratory, The Francis Crick Institute, 1 Midland Road, London, NW1 1AT, UK.
[2] Chromosome Replication Laboratory, The Francis Crick Institute, 1 Midland Road, London, NW1 1AT, UK.
[3] Structural Biology Science Technology Platform, The Francis Crick Institute, 1 Midland Road, London, NW1 1AT, UK.
[4] Department of Structural and Molecular Biology, Institute of Structural and Molecular Biology, University College London.
[5] Institute of Structural and Molecular Biology, Biological Sciences, Birkbeck College.

[§] Equal contribution.

[*]Corresponding Author
alessandro.costa@crick.ac.uk
Tel: +44 (0) 203 796 1812

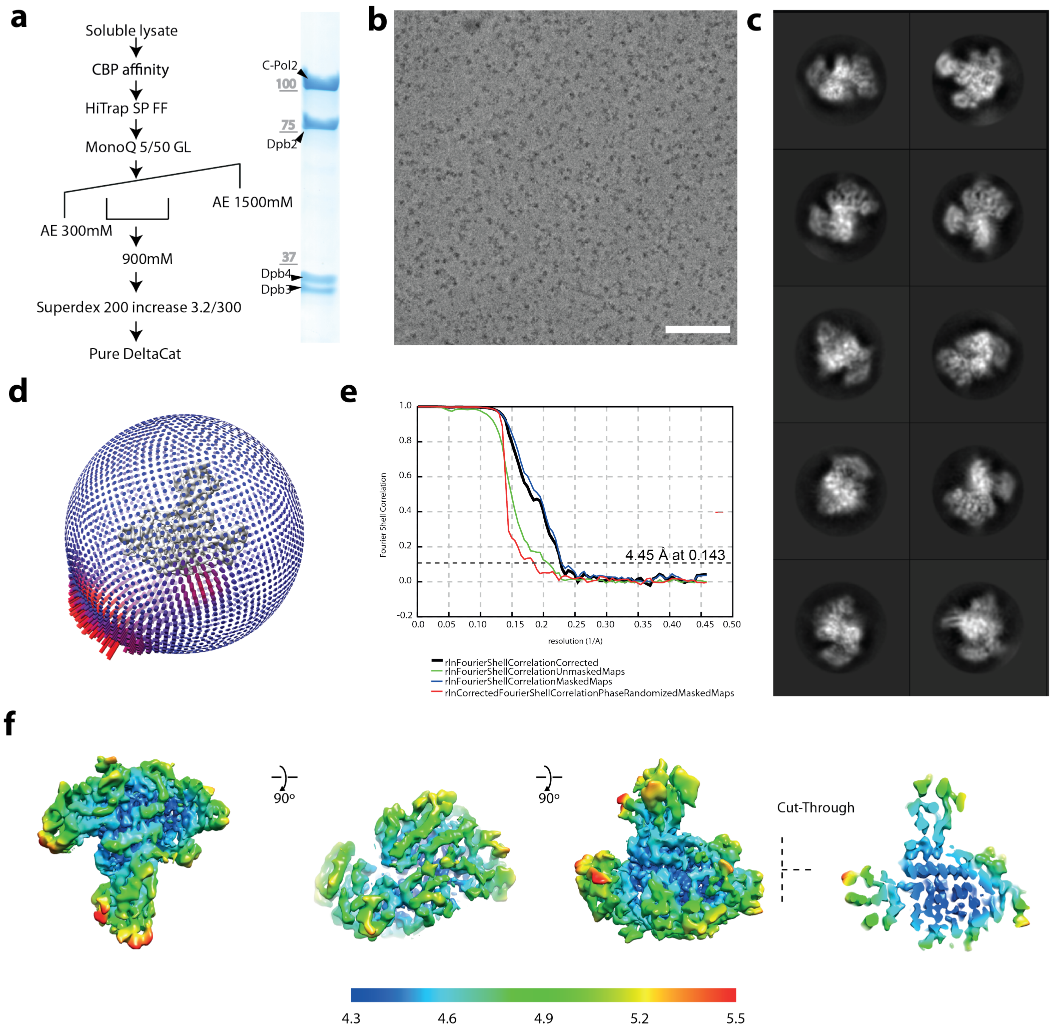

Supplementary Figure 1: Cryo-electron microscopy of the N-terminal Pol2 deletion (deltacat) variant of the tetrameric Pol epsilon complex. **(a)** Purification strategy and Coomassie-stained gel of deltacat. **(b)** Electron micrograph (aligned sum) acquired using a Volta phase plate on a Falcon III direct detector in counting mode. Scale bar 100 nm. **(c)** Representative 2D class averages. **(d)** Angular distribution. **(e)** Gold-standard Fourier shell correlatation and resolution estimation using the 0.143 criterion. **(f)** Cryo-EM map color-coded by local resolution.

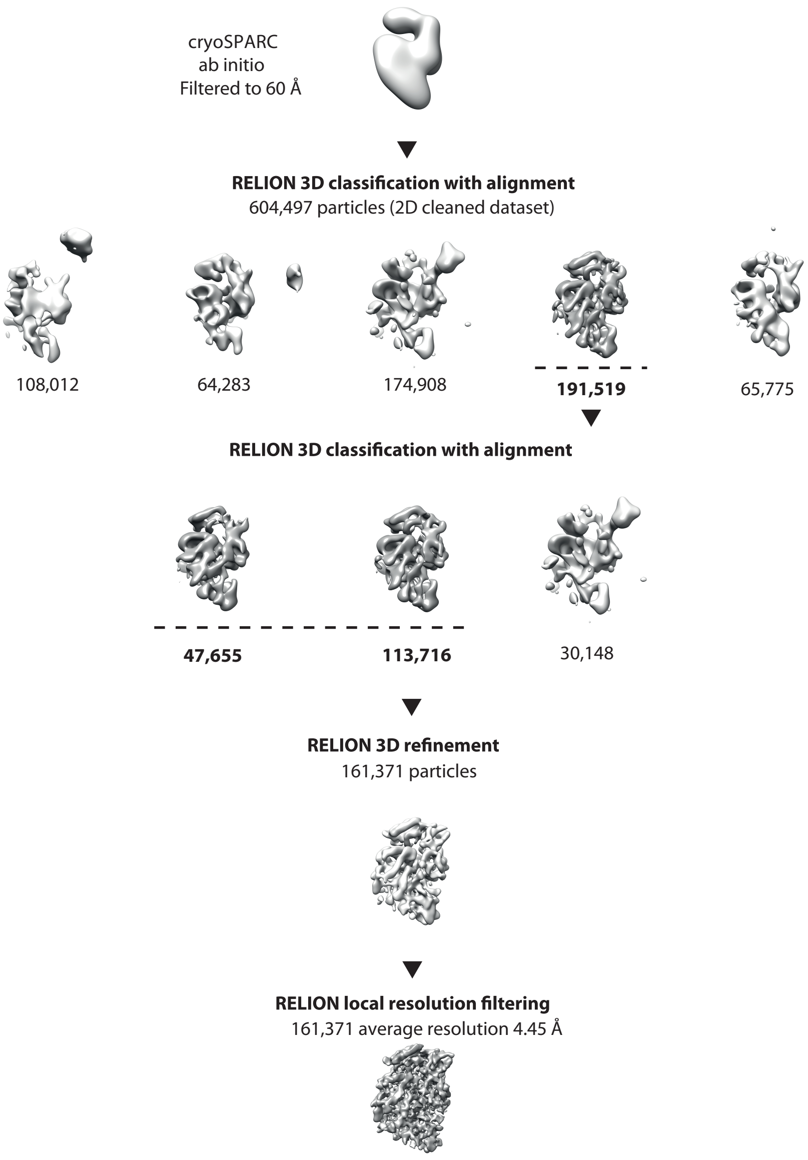

Supplementary Figure 2: Overview of image processing for deltacat.

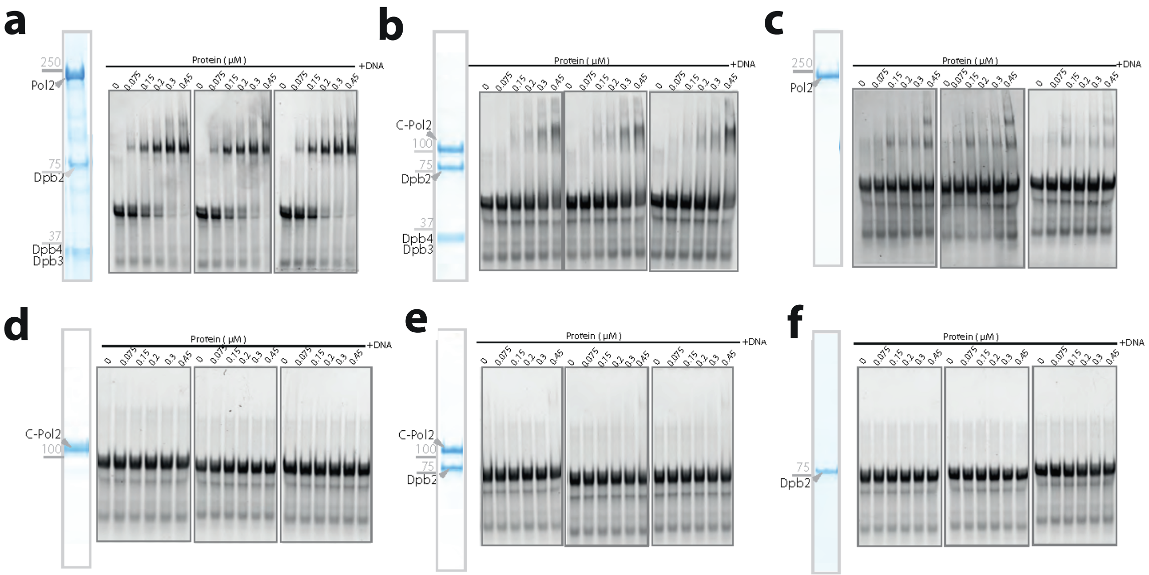

Supplementary Figure 3: Coomassie-stained gel of purified Polymerase epsilon and its dropout variants. Three repeats of the DNA binding assays visualized using the Diamond Nucleic Acid dye. **(a)** Wild type Pol epsilon **(b)** deltacat **(c)** Pol2 **(d)** C-Pol2 **(e)** C-Pol2-Dpb2 **(f)** Dpb2.

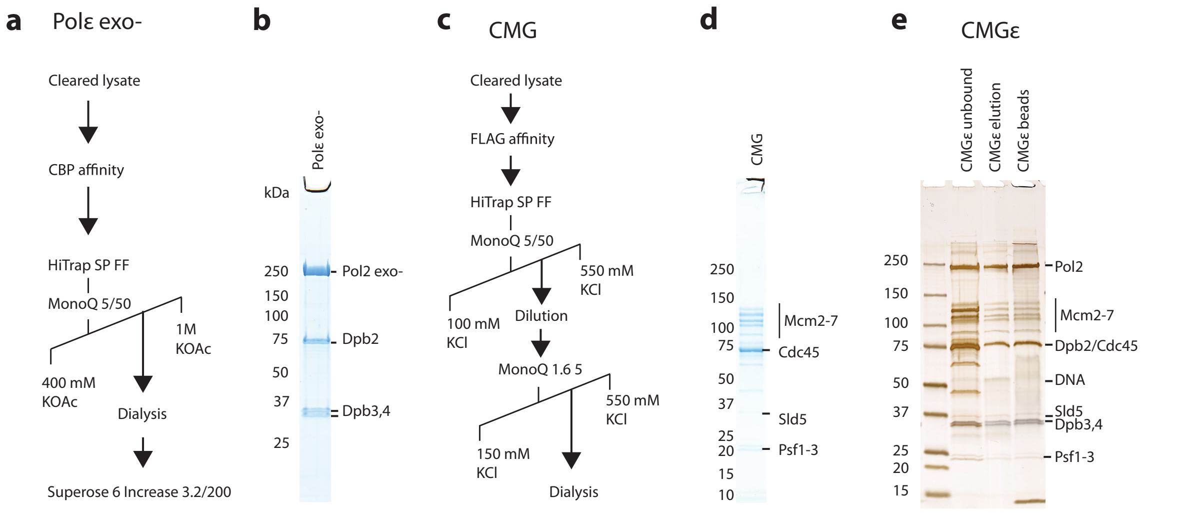

Supplementary Figure 4: Protein purification and reconstitution of DNA-bound CMG-Pol epsilon. **(a)** Purification strategy for full length Pol epsilon exo^{-}. **(b)** Coomassie-stained gel. **(c)** Purification strategy for CMG. **(d)** Coomassie-stained gel of purified CMG. **(e)** Silver stained gel of CMG-Polymerase epsilon exo- unbound, eluted and bead fractions.

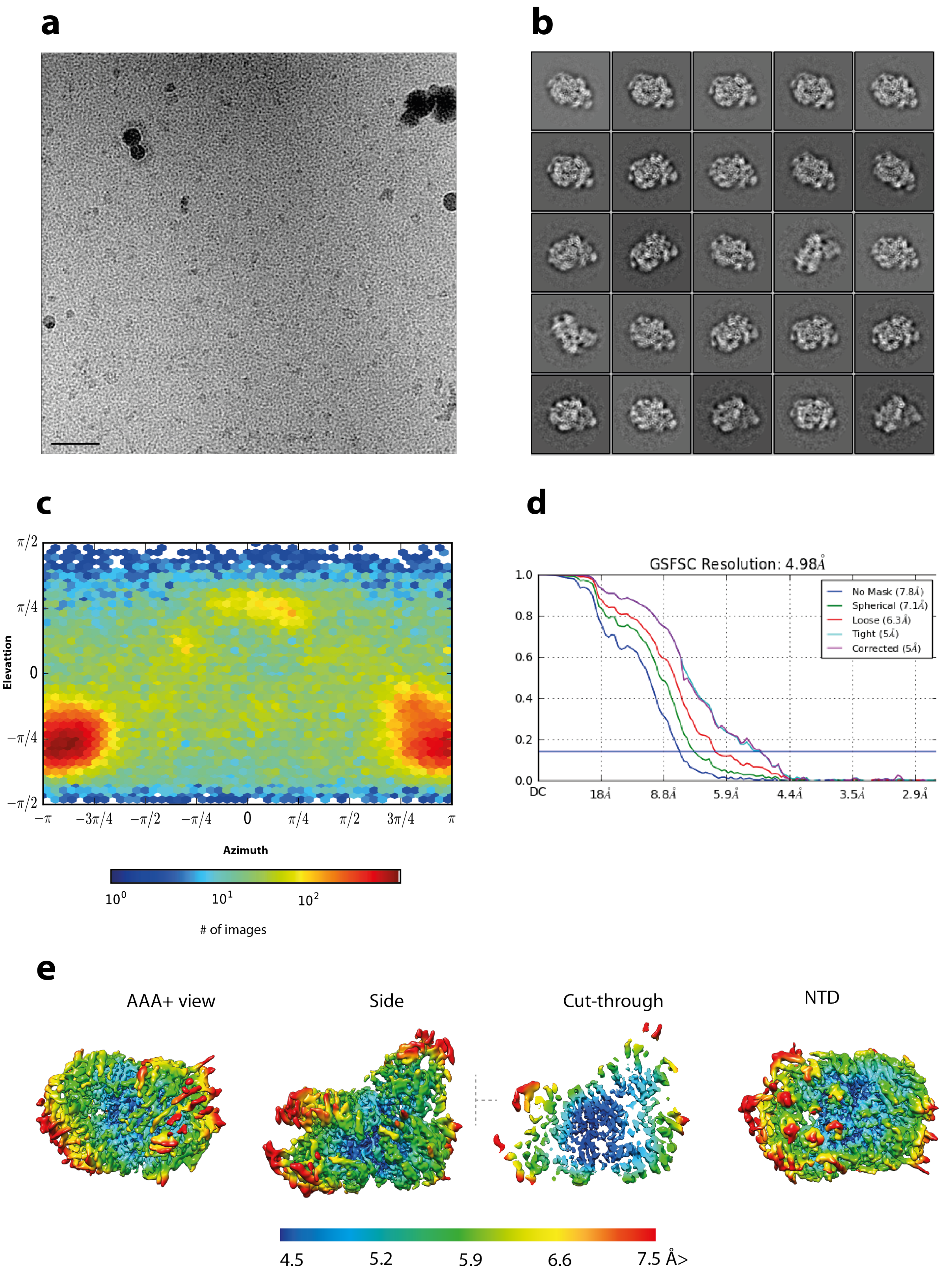

Supplementary Figure 5: Cryo-electron microscopy of the DNA-bound CMG Pol epsilon. **(a)** Cryo-electron micrograph acquired with a K2 summit camera operated in counting mode. Scale bar 100 nm. **(b)** Selection of 2D class averages after 3D refinement. **(c)** Angular distribution. **(d)** Gold standard Fourier shell correlatation using the 0.143 criterion. **(e)** Local resolution as determined by Relion.

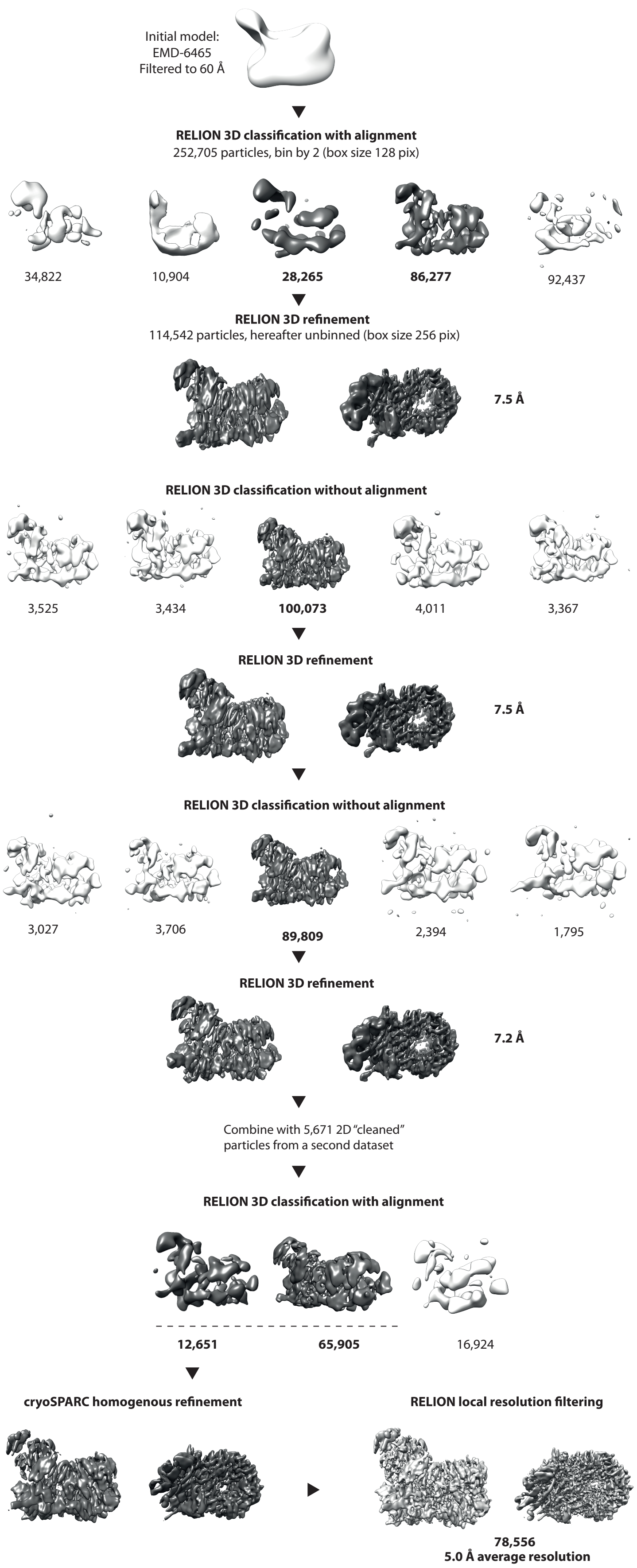

Supplementary Figure 6: Overview of image processing for the DNA-bound CMG-Pol epsilon.

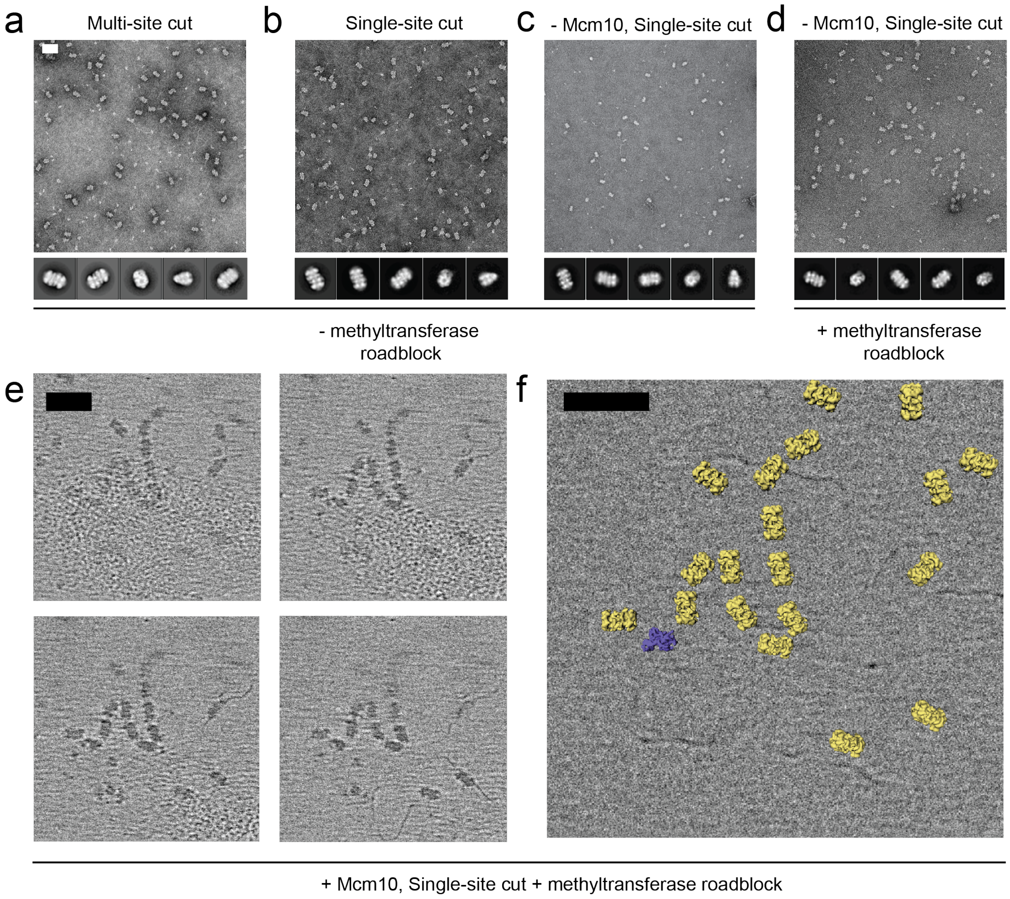

Supplementary Figure 7: Imaging helicase activation reconstituted in vitro using purified yeast proteins. **(a-d)** Stacks of MCM double hexamers ("MCM trains") cannot be observed if DNA does not contain a methyltransferase roadblock. This can be observed when DNA is digested with a multi-site restriction enzyme (a), with a single site cutter (b) and when Mcm10 activator of the CMG helicase is omitted (c). MCM trains cannot be visualised in the presence of the methyltransferase roadblock if the Mcm10 activator is omitted. Scale bar 50 nm. **(e)** Reconstructed cryo-electron tomogram (4 slicer views) showing one example of an MCM train. Scale bar 50 nm. **(f)** Template matching indicate that one stack of MCM double hexamers is capped at one end by one CMG-Pol epsilon complex. Scale bar 50 nm.

Supplementary Table 1: Yeast strains

Strain	Description	Genotype	Source
yAJ2	WT Polymerase epsilon	MATa ade2-1 ura3-1 his3-11,15 trp1-1 leu2-3,112 can1-100<br>bar1::Hyg<br>pep4::KanMX<br>TRP Gal1-10/ Pol2 + Dpb4-CBP<br>URA Gal1-10/ Dpb2 + Dpb3	Yeeles et al.<br>Nature. 2015
yAE99	Polymerase epsilon exo$^-$	MATaade2-1 ura3-1 his3-11,15 trp1-1 leu2-3,112 can1-100<br>bar1::Hyg<br>pep4::KanMX<br>ura3::URA3-Gal1-10/ Dpb2 + Dpb3<br>Pol2::Pol2-FLAG-NAT<br>trp1::TRP1-Gal1-10/ Pol2 exo- + Dpb4-CBP	This study<br>(exo- Pol2 contains mutation D290A, E292A) as in Morrison et al. PNAS 1991.
yAJ25	deltacat	MATa ade2-1 ura3-1 his3-11,15 trp1-1<br>leu2-3,112 can1-100<br>bar1::Hyg<br>pep4::KanMX<br>ura3::URA3-Gal1-10/ Dpb2 + Dpb3<br>trp1::TRP1-Gal1-10/ Pol2 Δ1–1262aa + Dpb4-TEV-CBP	Yeeles et al.<br>Mol Cell 2017
yAJ32	Pol2	MATa ade2-1 ura3-1 his3-11,15 trp1-1 leu2-3,112 can1-100<br>bar1::Hyg<br>pep4::KanMX<br>trp1::TRP1-Gal1-10/ Pol2–3x FLAG	This Study
yAJ30	C-Pol2	MATa ade2-1 ura3-1 his3-11,15 trp1-1 leu2-3,112 can1-100<br>bar1::Hyg<br>pep4::KanMX<br>trp1::TRP1-Gal1-10/ Pol2 Δ1–1262aa–3xFLAG	This Study
yAJ33	Dpb2	MATa ade2-1 ura3-1 his3-11,15 trp1-1 leu2-3,112 can1-100<br>bar1::Hyg<br>pep4::KanMX<br>ura3::URA3-Gal1-10/ Dpb2–3xFLAG	This Study
yAJ31	C-Pol2/Dpb2	MATa ade2-1 ura3-1 his3-11,15 trp1-1 leu2-3,112 can1-100<br>bar1::Hyg<br>pep4::KanMX<br>ura3::URA3-Gal1-10/ Dpb2<br>trp1::TRP1-Gal1-10/ Pol2 Δ1–1262aa–3xFLAG	This Study
yJCZ3	CMG	yAM22: MATa pep4::KanMx4<br>bar1::Hph-NT1<br>trp1-1::TRP1pJF3 (MCM5-GAL1-10-MCM4)<br>leu2-3::LEU2pJF4 (MCM7-GAL1-10-MCM6)<br>ura3-1::URA3pJF5 (MCM2-GAL1-10-CBP-TEV-MCM3)<br>yJCZ1: MATα pep4::KanMx4<br>bar1::Hph-NT1<br>his3-11::HIS3pJY13 ($CDC45^{iFLAG2}$-GAL1-10-GAL4)<br>trp1-1::TRP1pJCZ1 (PSF1-GAL1-10-SLD5)<br>leu2-3::LEU2pJCZ2 (PSF2-GAL1-10-PSF3)<br>ade2-1::pJCZ3 (ADE2).	Zhou et al PNAS 2017

Supplementary Table 2: Cryo-EM data collection, refinement and validation statistics

	deltacat Polε (EMDB-0287) (PDB 6HV8)	CMGPolε-DNA (EMD-0288) (PDB 6HV9)
**Data collection and processing**		
Nominal magnification	75,000	105,000
Voltage (kV)	300	300
Detector	Falcon III	K2 Summit
Frames	30	30
Electron exposure (e–/Å^2)	30	50
Defocus range (μm)	-0.5 to -1	-2.5 to -4.5
Pixel size (Å)	1.09	1.38
Symmetry imposed	C1	C1
Initial particle images (no.)	816,814	404,676
Final particle images (no.)	161,376	78,556
Map resolution (Å)	4.45	4.98
FSC threshold	(0.143)	(0.143)
Map resolution range (Å)	4.3-5	4.5-7.5
**Refinement**		
Initial model used (PDB code)	N/A	5u8s
Model resolution (Å)	4.40	4.50
FSC threshold	0.143	0.143
Model resolution range (Å)	N/A	60
Map sharpening *B* factor (Å^2)	-300	-300
R.m.s. deviations		
Bond lengths (Å)	0.009	0.012
Bond angles (°)	1.641	1.870
Validation		
MolProbity score	2.29	2.08
Clashscore	9	9.26
Poor rotamers (%)	1.1	0.48
Ramachandran plot		
Favored (%)	76.75	88.65
Allowed (%)	22.73	11.05
Disallowed (%)	0.52	0.29
