# Supplementary material for: Structure of DNA-CMG-Pol epsilon elucidates the roles of the non-catalytic polymerase modules in the eukaryotic replisome
Source: Nat Commun. 2018 Nov 29;9:5061. doi: 10.1038/s41467-018-07417-1 (PMC6265327; doi:10.1038/s41467-018-07417-1)
Supplement: Supplementary file 2 — Description of Additional Supplementary Information [file 41467_2018_7417_MOESM2_ESM.pdf]

**Description of Additional Supplementary Files**

File Name: Supplementary Movie 1
Description: Flexibility in the Pol epsilon deltacat complex. A structured module is flexibly tethered to the C-Pol2/Dpb2 core.

File Name: Supplementary Movie 2
Description: Atomic model of C-Pol2/Dpb2 built into Pol epsilon deltacat cryo-EM density.

File Name: Supplementary Movie 3
Description: Atomic model for DNA-CMG-Pol epsilon built into the cryo-EM map.

File Name: Supplementary Movie 4
Description: Conformational transitions between CMG and CMGE on DNA. CMG-DNA contains three nucleotide-occupied ATPase centers. These are Mcm5-3, Mcm2-5 and Mcm6-2. Sites can be seen occupied with AMP-PNP (in the single-stranded-DNA-bound CMG structure) or with ATP (in the CMG structure engaged to the duplex/single-stranded DNA junction). Morphing to the CMGE-ATPγS structure highlights tightening of Mcm5-3 and Mcm2-5 sandwiching an ATPγS molecule. The three MCM protomers are directly contacted by C-Pol2 and Dpb2 in the CMGE. Notably Mcm2-6 appears nucleotide-free. The Pol epsilon-driven conformational change in the CMG appears to promote a reconfiguration of fork nexus engagement.

File Name: Supplementary Movie 5
Description: Reconstructed cryo-electron tomogram showing trains of MCM double hexamers capped at one end by an advancing CMG-Pol epsilon complex. Double hexamers and the CMG complex are placed inside the cryotomogram using template matching.

File Name: Supplementary Movie 6
Description: Conformational variability in the apo CMG involve i. large-scale movements of the Mcm5 WH domain, ii. fluctuation between a spiral and a planar state of the ATPase tier and iii. opening/closing of the Mcm2-5 ATPase gate. By latching the Mcm2-5 gate and binding to the Mcm5 WH element, C-Pol2/Dpb2 limit flexibility of the ATPase module in the CMG.
