# Supplementary material for: Structure of DNA-CMG-Pol epsilon elucidates the roles of the non-catalytic polymerase modules in the eukaryotic replisome
Source: Nat Commun. 2018 Nov 29;9:5061. doi: 10.1038/s41467-018-07417-1 (PMC6265327; doi:10.1038/s41467-018-07417-1)
Supplement: Supplementary file 9 — Reporting Summary [file 41467_2018_7417_MOESM9_ESM.pdf]

nature research

Corresponding author(s): Alessandro Costa

☐ Initial submission ☐ Revised version ☒ Final submission

Life Sciences Reporting Summary

Nature Research wishes to improve the reproducibility of the work that we publish. This form is intended for publication with all accepted life science papers and provides structure for consistency and transparency in reporting. Every life science submission will use this form; some list items might not apply to an individual manuscript, but all fields must be completed for clarity.

For further information on the points included in this form, see Reporting Life Sciences Research. For further information on Nature Research policies, including our data availability policy, see Authors & Referees and the Editorial Policy Checklist.

Experimental design

1. Sample size

Describe how sample size was determined.	The sample size was determined based on the target near atomic resolution in the cryo-EM maps. -
Describe any data exclusions.	Micrographs containing limited resolution information were discarded. Particle contributing to low-resolution 2D and 3D averages were also discarded.
Replication Describe whether the experimental findings were reliably reproduced.	Experiments were highly reproducible. The same Pol epsilon deltacat structure were solved from 3 different datasets.
Describe how samples/organisms/participants were allocated into experimental groups.	Not relevant.

5. Blinding

Describe whether the investigators were blinded to group allocation during data collection and/or analysis.	Not relevant.

Note: all studies involving animals and/or human research participants must disclose whether blinding and randomization were used.

6. Statistical parameters

For all figures and tables that use statistical methods, confirm that the following items are present in relevant figure legends (or in the Methods section if additional space is needed).

n/a	Confirmed	
☐	☒	The exact sample size (n) for each experimental group/condition, given as a discrete number and unit of measurement (animals, litters, cultures, etc.)
☐	☒	A description of how samples were collected, noting whether measurements were taken from distinct samples or whether the same sample was measured repeatedly
☐	☒	A statement indicating how many times each experiment was replicated
☐	☒	The statistical test(s) used and whether they are one- or two-sided (note: only common tests should be described solely by name; more complex techniques should be described in the Methods section)
☐	☒	A description of any assumptions or corrections, such as an adjustment for multiple comparisons
☐	☒	The test results (e.g. P values) given as exact values whenever possible and with confidence intervals noted
☐	☒	A clear description of statistics including central tendency (e.g. median, mean) and variation (e.g. standard deviation, interquartile range)
☐	☒	Clearly defined error bars

See the web collection on statistics for biologists for further resources and guidance.

Software

Policy information about availability of computer code

7. Software

Describe the software used to analyze the data in this study.

Established packages for single-particle cryo-EM reconstruction were used here, including Relion2.1 and cryoSPARC.

For manuscripts utilizing custom algorithms or software that are central to the paper but not yet described in the published literature, software must be made available to editors and reviewers upon request. We strongly encourage code deposition in a community repository (e.g. GitHub). *Nature Methods* guidance for providing algorithms and software for publication provides further information on this topic.

Materials and reagents

Policy information about availability of materials

8. Materials availability

Indicate whether there are restrictions on availability of unique materials or if these materials are only available for distribution by a for-profit company.

No restriction.

9. Antibodies

Describe the antibodies used and how they were validated for use in the system under study (i.e. assay and species).

We used an anti-Psf1-3 antibody that was validated against recombinant yeast proteins.

10. Eukaryotic cell lines

a. State the source of each eukaryotic cell line used.

S. cerevisiae overexpression strains described in Frigola et al 2013 amd On et al 2014.

b. Describe the method of cell line authentication used.

-

c. Report whether the cell lines were tested for mycoplasma contamination.

-

d. If any of the cell lines used are listed in the database of commonly misidentified cell lines maintained by ICLAC, provide a scientific rationale for their use.

-

Animals and human research participants

Policy information about studies involving animals; when reporting animal research, follow the ARRIVE guidelines

11. Description of research animals

Provide details on animals and/or animal-derived materials used in the study.

-

Policy information about studies involving human research participants

12. Description of human research participants

Describe the covariate-relevant population characteristics of the human research participants.

-
